# Nonnegative matrix factorization incorporating domain specific constraints for four dimensional scanning transmission electron microscopy

**DOI:** 10.1038/s41598-025-23541-7

**Published:** 2025-11-07

**Authors:** Koji Kimoto, Fumihiko Uesugi, Koji Harano, Jun Kikkawa, Ovidiu Cretu, Yuki Shibazaki, Motoki Shiga, Atsushi Togo

**Affiliations:** 1 Center for Basic Research on Materials, National Institute for Materials Science, 1-1 Namiki, Tsukuba, Ibaraki 305-0044 Japan; 2 Research Network and Facility Services Division, National Institute for Materials Science, Tsukuba, Japan; 3 Research Center for Autonomous Systems Materialogy, Institute of Science Tokyo, Yokohama, Japan; 4 Institute of Materials Structure Science, High Energy Accelerator Research Organization, Tsukuba, Japan; 5 Unprecedented-scale Data Analytics Center, Tohoku University, Sendai, Japan; 6 Graduate School of Information Sciences, Tohoku University, Sendai, Japan; 7 Center for Advanced Intelligence Project, RIKEN, Tokyo, Japan

**Keywords:** Unsupervised machine learning, Scanning transmission electron microscopy (STEM), Nonnegative matrix factorization (NMF), Principal component analysis (PCA), Clustering, Hyperspectral imaging, Microscopy, Transmission electron microscopy

## Abstract

Modern electron microscopy enables the acquisition of extremely large datasets, necessitating optimized machine learning techniques, such as dimensionality reduction and clustering, to extract material insights. We propose a novel nonnegative matrix factorization (NMF) technique that integrates domain-specific constraints inherent to electron microscopy, including spatial resolution and continuous intensity features without downward-convex peaks. This constrained NMF was applied to four-dimensional (4D) scanning transmission electron microscopy (STEM). Using the constrained NMF, both simulated and actual experimental data were successfully decomposed into interpretable diffractions and maps that cannot be achieved using principal component analysis (PCA) and primitive NMF methods. Additionally, hierarchical clustering was optimized based on diffraction similarity, which is a combination of a polar coordinate transformation and uniaxial cross-correlation. Then, nanometer-sized crystalline precipitates embedded in an amorphous metallic glass, ZrCuAl, were successfully detected and classified according to their diffraction patterns. The present scheme is broadly applicable across various characterization techniques, including hyperspectral imaging, and effectively mitigates the known artifacts found in conventional machine learning techniques that rely solely on mathematical constraints without domain-specific knowledge.

**Supplementary Information:**

The online version contains supplementary material available at 10.1038/s41598-025-23541-7.

## Introduction

**Fig. 1:**
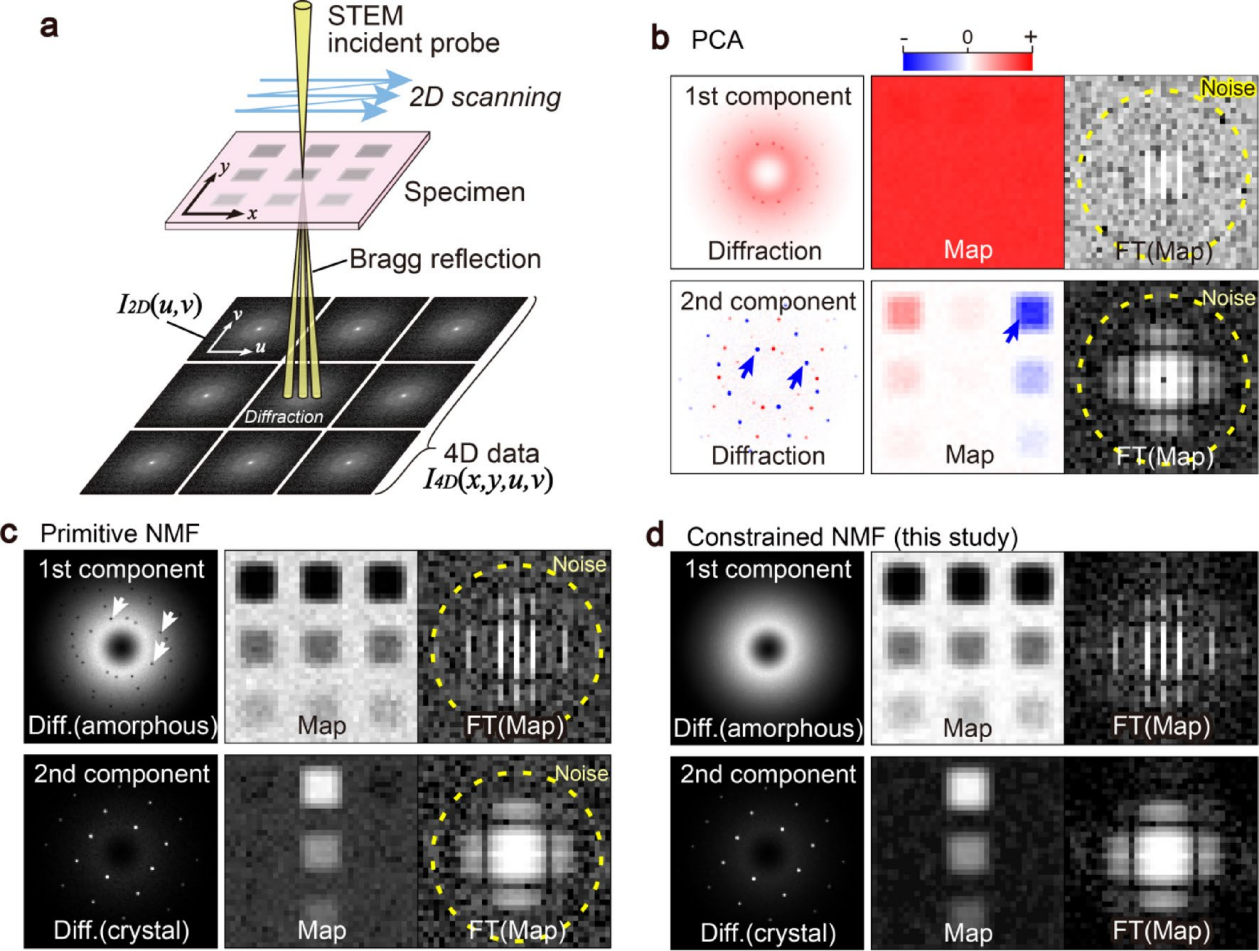
Schematic drawings of Four-dimensional scanning transmission electron microscopy (4D-STEM) and various factorizations. Factorized results include the first and the second diffractions and corresponding maps. Fourier transforms (FTs) of the maps are also shown. **a** Schematic of 4D-STEM, **b** Principal component analysis (PCA) of simulated 4D-STEM data. Blue arrows indicate artifacts of negative intensities. Dotted lines in the FTs of the maps indicate the resolution limits, and their outer parts represent high-frequency noise. **c** Primitive nonnegative matrix factorization (NMF). White arrows indicate artifacts of downward-convex peaks. **d** NMF with domain-specific constraints. These results do not show artifacts, such as downward-convex peaks in the diffractions or high-frequency noise in the maps.

 Modern scientific instruments generate significantly larger datasets than previous ones. Four-dimensional scanning transmission electron microscopy 4D-STEM^1–5^ is an advanced electron microscopy technique in which two-dimensional (2D) electron diffractions ***I***_***2D***_(*u*,* v*) are acquired by the STEM incident probe at varying positions (*x*, *y*), where (*u*,* v*) and (*x*,* y*) are the reciprocal and real-space coordinates, respectively (Fig. 1a). 4D-STEM provides bimodal information from both real and reciprocal spaces as maps and diffractions. This technique yields extensive 4D data, ***I***_***4D***_(*x*,* y*,*u*,* v*), and can be regarded as the basis of all STEM imaging techniques, including ptychography and differential phase contrast.

The significantly larger datasets from these scientific instruments necessitate integrating various machine learning techniques to extract meaningful material insights^6–9^. Well-established machine learning tools, such as scikit-learn^10^, HyperSpy^11^DSTEM^1^, and MATLAB, have already significantly benefited materials science, and machine learning techniques have been applied to 4D-STEM several times^12–18^. Dimensionality reduction techniques in unsupervised machine learning are indispensable tools for materials characterization, with principal component analysis (PCA)^19–22^ and nonnegative matrix factorization (NMF)^23–25^ being commonly used. Both PCA and NMF can approximately describe larger datasets using the matrix products of low-rank matrices.

However, although established tools have been applied to various research domains as they are, they do not integrate domain-specific knowledge (e.g., the properties of scientific instrumentation) into the machine learning algorithms themselves. Consequently, these tools can provide factorized components that cannot be interpreted as scientific measurement signals. A typical example is a negative intensity in the PCA, as shown in Fig. 1b, whereas electron microscopy signals physically must be positive because the number of detected electrons cannot be negative. Although NMF is used to circumvent the negative intensities, it still shows physically implausible artifacts. Here, we focus on implementing domain-specific knowledge in electron microscopy, such as resolution and intensity profiles. Various types of resolution (spatial, angular, or energy) must be improved; however, these resolutions can also be used as constraints to distinguish noise from signals through smoothing or Fourier filtering. Additionally, experimental results often show continuous intensity profiles for various reasons, which can be modeled based on physics but are not always represented by mathematical constraints. Conventional NMF can provide physically unrealistic high-frequency information or downward-convex peaks in a continuous intensity profile (Fig. 1c).

In this study, we propose a novel scheme to perform constrained NMF that incorporates knowledge of electron microscopy, such as the resolution in maps and intensity profile characteristics in diffractions (Fig. 1d). We applied the constrained NMF to both simulated and experimental 4D-STEM data (see the Methods). Given its foundation in resolution and intensity profile constraints, which are common across scientific instruments, the proposed scheme can be adapted for other hyperspectral imaging techniques.

## Results

### Outlines of primitive NMF algorithms and 4D-STEM

This section briefly summarizes the basics of primitive NMF and its combination with 4D-STEM. Advanced reports on NMF algorithms^26,27^, a textbook by pioneering researchers^28^, a comprehensive review of chemometrics^29^, and a modern textbook^30^ have been published. The experimental data, the matrix ***X***, are approximated by the product of lower-rank matrices ***W*** and ***H*** consisting of positive elements:

1 $$\:\varvec{X}\cong\:\varvec{W}\varvec{H},$$

where ***W*** and ***H*** denote the basis and their coefficients, respectively. In many NMF applications (e.g., object recognition^26^ and text mining), the sequence of the column vectors of ***X*** is irrelevant. In scientific measurements, however, the sequence of the column vectors in ***X*** corresponds to the position (e.g., hyperspectral imaging) or time (e.g., acoustic analysis). Although the matrix ***X*** can be transposed in its definition, in this study, the rows and columns of ***X*** represent the reciprocal (spectral) and real-space coordinates of 4D-STEM, respectively.

We determine the low-rank matrices ***W*** and ***H*** by minimizing a cost function *D* (an objective function), based on the Frobenius norm $\:\left\| {\: \cdot \:} \right\|_{F} \:$of the error as follows:

2 $$D(\varvec{X}||\varvec{WH}) = \frac{1}{2}\left\| {\varvec{X} - \varvec{WH}} \right\|_{F} ^{2} .$$

Two major algorithms can minimize the cost function in Eq. (2) using iterative procedures: (a) multiplicative update (MU) or (b) alternating least squares (ALS). In the case of the MU algorithm, the following update equations are used based on the majorization–minimization flamework^26^:

3 $$\:\varvec{W}\leftarrow\:\varvec{W}{ \circledast }\varvec{X}{\varvec{H}}^{\varvec{T}}\oslash\:\varvec{W}\varvec{H}{\varvec{H}}^{\varvec{T}},$$

4 $$\:\varvec{H}\leftarrow\:\varvec{H} { \circledast } {\varvec{W}}^{\varvec{T}}\varvec{X}\oslash\:{\varvec{W}}^{\varvec{T}}\varvec{W}\varvec{H},$$

where ${ \circledast }$ and $\:\oslash\:$ denote elementwise multiplication and division, respectively.

Alternatively, the ALS algorithm can be performed using the following equations:

5 $$\:\varvec{W}\leftarrow\:{\left[\left(\varvec{X}{\varvec{H}}^{\varvec{T}}\right){\left(\varvec{H}{\varvec{H}}^{\varvec{T}}\right)}^{-1}\right]}_{+},$$

6 $$\:\varvec{H}\leftarrow\:{\left[{\left({\varvec{W}}^{\varvec{T}}\varvec{W}\right)}^{-1}\left({\varvec{W}}^{\varvec{T}}\varvec{X}\right)\right]}_{+},$$

where [$\:\cdot\:$]_+_ represents a nonnegativity constraint projection^28^, i.e., $\:{\left[\varvec{W}\right]}_{+}=\text{m}\text{a}\text{x}\left\{\bf{0},\varvec{W}\right\}$. Because the ALS algorithm implements a constraint as a projection, domain-specific constraints can be flexibly designed.

Both MU and ALS algorithms are standard NMF solvers in the established tools, e.g., MATLAB (‘mult’ and ‘als’ of the function nnmf()) and scikit-learn (‘mu’ and ‘cd’ of NMF()). It is pointed out^30^ that the ALS algorithm is not mathematically rigorous, particularly for the projection [$\:\cdot\:$]_+_ onto the nonnegative orthant. In this study, we utilized both the MU and ALS algorithms and monitored their convergence, as discussed below. In the Supplementary Information, we provide usable scripts for DigitalMicrograph (Gatan Inc.)^31^ for both the MU and ALS algorithms as Listings S1 and S2, respectively.

Primitive NMFs with either algorithm tend to yield sparse components^32^; however, the sparse components are not always interpretable based on the physics of electron microscopy. If the actual components are not sparse, primitive NMF produces physically implausible sparse components. For example, if an experimental result consists of a continuous intensity (e.g., baseline) and additional sharp peaks, which are common in scientific measurements, primitive NMF inserts downward-convex peaks into the continuous intensity (e.g., white arrows of Fig. 1c), which is a known artifact as the unnatural drop in intensity^16,25,33^. In addition, primitive NMF does not implement 2D frequency analysis and cannot discriminate high-frequency noise. In the case of noisy datasets, it may provide high-frequency components that are physically impossible, and the nonnegativity constraint alone cannot solve these issues. In the following sections, we discuss two constraints related to the resolution and intensity profile features of scientific measurements.

**Fig. 2:**
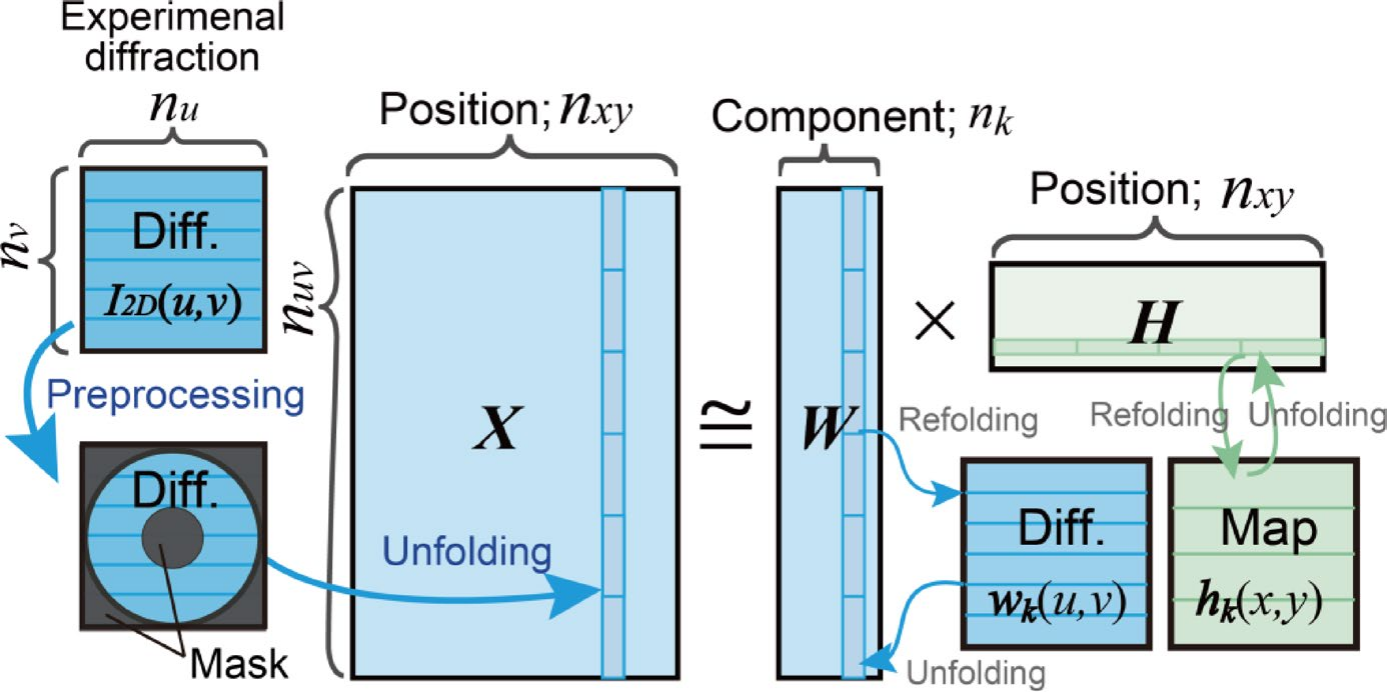
Schematic drawing of the matrix calculation for 4D-STEM.

To apply NMF to 4D-STEM, 4D data $\:{\varvec{I}}_{4\varvec{D}}\left(x,y,u,v\right)$ must be transformed into the matrix $\:\varvec{X}$. We transform the 2D experimental diffractions ***I***_***2D***_(*u*,* v*) into one-dimensional (1D) column vectors of the matrix ***X***, such that the rows and columns of ***X*** represent the reciprocal and real-space coordinates, respectively (Fig. 2). If the data point of each coordinate (*x*,* y*, *u*,* v*) in the 4D data is $\:\left({n}_{x},{n}_{y},{n}_{u},{n}_{v}\right)$ and the assumed number of components in NMF is *n*_*k*_ (*n*_k_ < < *n*_*xy*_), then $\:\varvec{X}\in\:{\mathbb{R}}_{+}^{{n}_{uv}\times\:{n}_{xy}}$, $\:\varvec{W}\in\:{\mathbb{R}}_{+}^{{n}_{uv}\times\:{n}_{k}}$, and $\:\varvec{H}\in\:{\mathbb{R}}_{+}^{{n}_{k}\times\:{n}_{xy}}$, where $\:{n}_{xy}={n}_{x}{n}_{y}$ and $\:{n}_{uv}={n}_{u}{n}_{v}$. Because the rows of ***W*** and the columns of ***H*** correspond to the reciprocal and real-space coordinates, respectively, they are referred to as the diffraction matrix ***W*** and the map matrix ***H*** in this study. NMF yields the assumed number of diffractions $\:{\varvec{w}}_{\varvec{k}}\left(u,v\right)$ and maps $\:{\varvec{h}}_{\varvec{k}}\left(x,y\right)$ (*k* = 0, 1, …, *n*_*k*_−1) as the *k*-th column and row vectors of ***W*** and ***H***, respectively. The transformation from a 1D vector into 2D data (maps and diffractions) and the reverse process are referred to as refolding (or reshaping) and unfolding, respectively. The dimensionality is reduced because the number of components *n*_*k*_, which is the column number of the matrix ***W***, is assumed to be smaller than the total number of experimental diffractions *n*_*xy*_.

As the cost function in Eq. (2), i.e., the Frobenius norm, is invariant to the multiplication of the permutation matrix, the sequences of columns and rows of ***X*** are arbitrary and irrelevant for primitive NMF calculations. In other words, the diffractions are processed as 1D vectors without probe position information, and various material characteristics (e.g., the spatial distribution in real space or the diffraction angle in reciprocal space) are not addressed. Thus, the bimodal information in 4D-STEM cannot be utilized in primitive NMF. This study aims to integrate such bimodal information into the NMF algorithm by applying constraints to 2D maps and diffractions.

### Protocol of constrained NMF with 4D-STEM knowledge

**Fig. 3:**
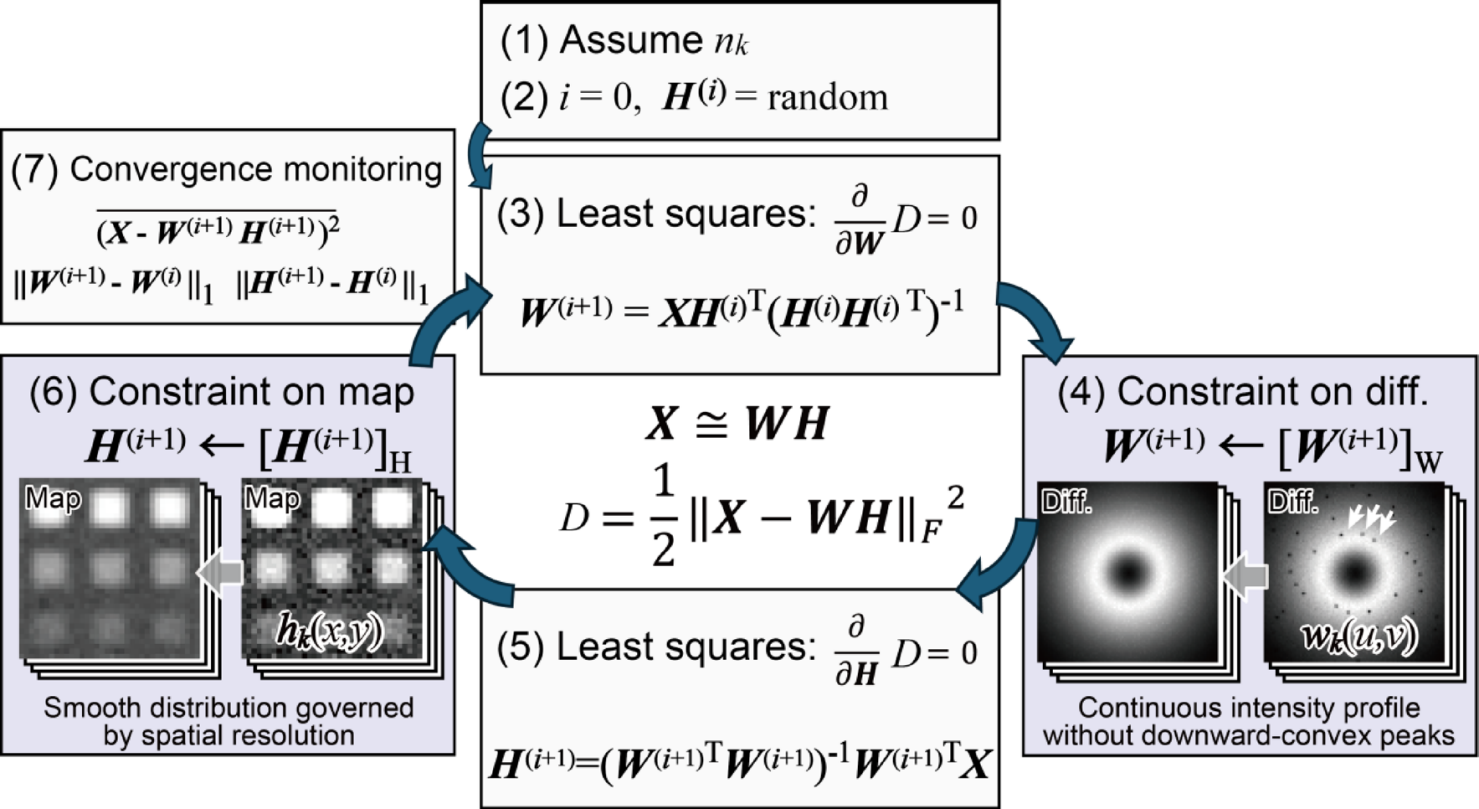
Schematic drawing of NMF with constraints on diffractions and maps. Domain-specific constraints $\:{[\:\cdot\:]}_{\text{W}}$ and $\:{[\:\cdot\:]}_{\text{H}}$ on diffractions and maps, respectively, are implemented in Steps (4) and (6), respectively.

The proposed NMF protocol can be classified as an ALS algorithm because Eqs. (7) and (8) correspond to the least-squares solutions derived from $\:\frac{\partial\:}{\partial\:\varvec{W}}D=0$ and $\:\frac{\partial\:}{\partial\:\varvec{H}}D=0$, respectively. The protocol consists of the following steps, as illustrated schematically in Fig. 3.

1. The number of components *n*_*k*_ is assumed.
2. The matrix $\:{\varvec{H}}^{\left(i\right)}$ is generated with its elements being nonnegative random numbers, where *i* represents the index of iterations.
3. 7 $${\varvec{W}}^{(i+1)}=\left(\varvec{X}\:{{\varvec{H}}^{\left(i\right)}}^{\text{T}}\right){\left({\varvec{H}}^{\left(i\right)}\:{{\varvec{H}}^{\left(i\right)}}^{\text{T}}\right)}^{-1}.$$
4.  A constraint on diffraction is applied, i.e., nonnegativity $\:{\left[{\varvec{W}}^{(i+1)}\right]}_{+}$or domain-specific $\:{\left[{\varvec{W}}^{(i+1)}\right]}_{\text{W}};$ then, $\:{\varvec{W}}^{(i+1)}\leftarrow\:{\left[{\varvec{W}}^{(i+1)}\right]}_{+}$ or $\:{\varvec{W}}^{(i+1)}\leftarrow\:{\left[{\varvec{W}}^{(i+1)}\right]}_{\text{W}}$. The details of the domain-specific constraints are explained later. Each column vector of $\:{\varvec{W}}^{(i+1)}$ is normalized.
5.  
  8 $$\:\:\:\:{\varvec{H}}^{(i+1)}={\left({{\varvec{W}}^{\left(i+1\right)}}^{\text{T}}{\:\varvec{W}}^{\left(i+1\right)}\right)}^{-1}\left({{\varvec{W}}^{\left(i+1\right)}}^{\text{T}}\:\varvec{X}\right).$$
6. A constraint on maps is applied, i.e., nonnegativity $\:{\left[{\varvec{H}}^{(i+1)}\right]}_{+}$ or domain-specific $\:{\left[{\varvec{H}}^{(i+1)}\right]}_{\text{H}}$; then, $\:{\varvec{H}}^{(i+1)}\leftarrow\:{\left[{\varvec{H}}^{(i+1)}\right]}_{+}$ or $\:{\varvec{H}}^{(i+1)}\leftarrow\:{\left[{\varvec{H}}^{(i+1)}\right]}_{\text{H}}$.
7. The mean squared error (MSE) $\:\overline{{{(\varvec{X}-{\varvec{W}}^{(i+1)}{\varvec{H}}^{(i+1)})}^{2}}}$ and the *L*_*1*_-norms of the differences $\:\left\| {\varvec{W}^{{(i + 1)}} - \varvec{W}^{{\left( i \right)}} } \right\|_{1}$ and $\left\| {\varvec{H}^{{(i + 1)}} - \varvec{H}^{{\left( i \right)}} } \right\|_{1}$ are calculated to monitor their convergence. The protocol then returns to Step (3) until the index of the iterations reaches a preset value (500 in this study).
8. To survey the global minimum, NMF is performed multiple times (ten in this study) from Steps (2) to (7), and the optimum matrices ***W*** and ***H*** are selected.
9. To place the major components first in the ***H*** rows and ***W*** columns, the row vectors of ***H*** are sorted according to their *L*_*2*_-norms, and the column vectors of ***W*** are sorted according to the order of the corresponding row vectors of ***H***.

Steps (4) and (6) primarily implement the nonnegativity constraints $\:{[\:\cdot\:]}_{+}$; however, additional domain-specific constraints have been introduced in this study. Two different domain-specific constraints on diffractions and maps are applied, which are denoted as $\:{[\:\cdot\:]}_{\text{W}}$ and $\:{[\:\cdot\:]}_{\text{H}}$, respectively. The former eliminates downward-convex peaks using rotational symmetry, and the latter reduces high-frequency noise by convolution with a kernel estimated from the spatial resolution (Fig. 3). The following section discusses these additional constraints in further detail. These constraints are stricter than conventional nonnegativity. Although the MSE is proportional to the cost function Eq. (2), we found that the MSE becomes inadequate for monitoring the convergence of iterations when these additional constraints are introduced, as discussed later (Fig. 7). We also calculated the *L*_*1*_-norms of the iterative differences in matrices ***W*** and ***H*** to monitor the convergence in Step (7).

**Fig. 4:**
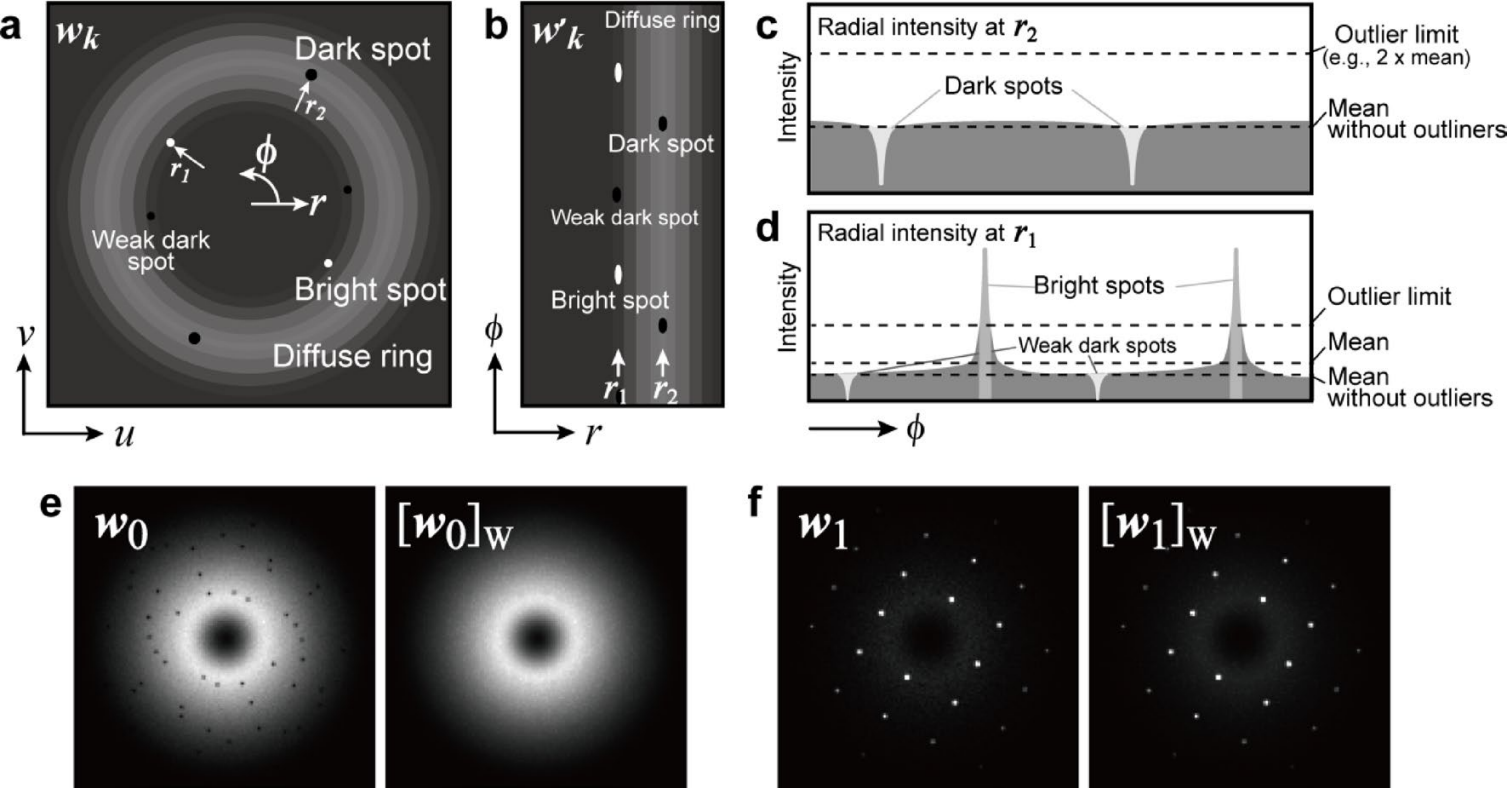
Schematics of the constraint on diffractions $\:{[\:\cdot\:]}_{\text{W}}$ with examples. **a** Schematic of the factorized diffraction $\:{\varvec{w}}_{\varvec{k}}\left(u,v\right)$, including diffraction spots (bright spots), an amorphous diffuse ring, and artifacts (dark spots). **b** *r*–*φ* transformed diffraction $\:{\varvec{w}\varvec{^{\prime}}}_{\varvec{k}}(r,{\varphi})$. Arrows *r*_*1*_ and *r*_*2*_ indicate the positions of the radial intensity profiles. **c** and **d** Radial intensity profiles **c** at *r*_*2*_ in which dark spots are included and **d** at *r*_*1*_ in which bright spots are included. **e** and **f** Examples of the constrained diffractions, which are processed by the proposed script (Listing S4).

### Constraint on diffractions $\:{[\:\cdot\:]}_{\mathbf{W}}$: continuous intensity feature

Scientific measurement data often show continuous intensity features for various reasons, such as background noise from a detection instrument or an actual signal (e.g., the baseline) originating from physical phenomena. In the latter case, the continuous intensity itself constitutes the material information of interest. Here, we consider the electron diffractions of 4D-STEM based on kinematical scattering theory, where the amplitudes of the diffraction scatterings are the product of the atomic scattering factors and the Laue function^34^. The atomic scattering factor decreases monotonically with the scattering angle. By contrast, the Laue function, which depends on the atomic arrangement, produces discrete peaks, particularly for single crystals, resulting in diffraction spots. In the case of an amorphous structure, it results in concentric diffuse rings. When amorphous and crystalline materials are mixed, the diffraction pattern becomes a sum of both crystalline and amorphous patterns, resulting in spots with concentric diffuse rings. The amorphous diffuse rings must have rotational symmetry; however, the amorphous components derived using primitive NMF show dark spots as artifacts (see Fig. 1c), similar to the unnatural intensity drop observed in hyperspectral imaging^16,25,33^. We thus leverage this rotational symmetry in the diffuse rings as a constraint to avoid an unnatural intensity drop in 4D-STEM.

Figure 4 schematically illustrates the procedure for applying the constraint to diffractions. The initial step is to refold the *n*_*k*_ column vectors of the matrix $\:{\varvec{W}}^{\left(i\right)}$ into a set of 2D diffractions $\:{{\varvec{w}}_{\varvec{k}}}^{\left(i\right)}\left(u,v\right)$, where *k* = 0, 1, …, *n*_*k*_−1 of each iteration *i*. As shown in Fig. 4a, the factorized diffractions comprise bright crystalline spots (Bragg spots), amorphous diffuse rings, and dark spot artifacts. Subsequently, each diffraction $\:{{\varvec{w}}_{\varvec{k}}}^{\left(i\right)}\left(u,v\right)$ is transformed into $\:{{\varvec{w}\varvec{{^\prime}}}_{\varvec{k}}}^{\left(i\right)}(r,{\varphi})$ (Fig. 4,b), i.e., a polar coordinate transformation. The radial intensity at each radius is derived from each column of the transformed diffraction. As illustrated in radial intensity at *r*_*2*_ (Fig. 4c), the dark spots can be identified as regions below the radial mean intensity; these regions are then substituted with the mean intensity, thereby eliminating the dark spots. Figure 4d shows the radial intensity at *r*_*1*_, which includes bright diffraction spots. To estimate the continuous intensity baseline, the bright spots must be treated as outliers larger than a certain threshold, which is assumed to be twice the radial mean intensity in this study. Subsequently, the radial mean intensity is recalibrated without the outliers. If weak dark spots appear at the same radius *r*_*1*_, these dark spots can be corrected to the recalibrated value. The processed $\:r-\varphi\:$ diffractions are then transformed back into 2D diffractions $\:{\left[{{\varvec{w}}_{\varvec{k}}}^{\left(i\right)}\left(u,v\right)\right]}_{\text{W}}$, and finally, they are unfolded into the column vectors of the diffraction matrix $\:{\varvec{W}}^{\left(i\right)}$. These processes are applied to each radius (e.g., *r*_*0*_, *r*_*1*_, *r*_*2*_, …, *n*_*u*_/2) of each diffraction component (*k* = 0,1, …, *n*_*k*_−1) in each iteration (*i* = 0,1, …, 499) of Step (4). All of these processes are represented as $\:{\varvec{W}}^{\left(i\right)}\leftarrow\:{\left[{\varvec{W}}^{\left(i\right)}\right]}_{\text{W}}$. Notably, all factorized components, including amorphous and crystalline diffractions, are processed equally using the same script; therefore, it is an unsupervised process. Figure 4e and f show examples of the constraint on diffractions. A DigitalMicrograph script for this constraint is provided in full in Sect. 3 of the Supplementary Information (Listing S4).

**Fig. 5:**
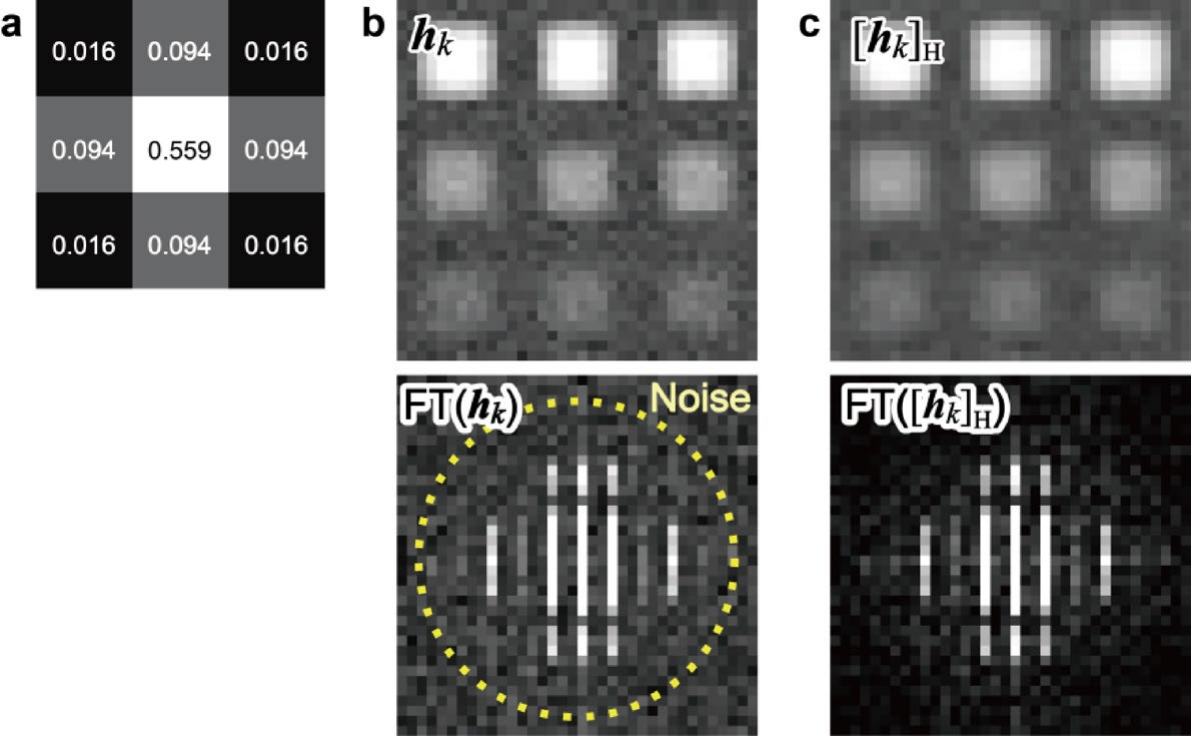
Procedure for the constraint on maps $\:{[\:\cdot\:]}_{\text{H}}$. **a** Convolution kernel for map. **b** and **c** Examples of a map **b** before and **c** after the convolution and its Fourier transform.

### Constraint on maps $\:{[\:\cdot\:]}_{\mathbf{H}}$: smoothness governed by spatial resolution

Resolution is the most critical parameter limiting the information that can be obtained in hyperspectral imaging. Although there are spatial and angular resolutions in 4D-STEM, we focus on the former here. The spatial resolution in STEM primarily depends on the incident probe size, and the experimental scanning step is often smaller than the probe, leading to oversampling. The spatial resolution determines the limit on the obtainable real-space information, and the observable frequency can be evaluated using the Fourier transform. Based on its spatial resolution, we can constrain the frequency limit as the obtainable information. An inverse Fourier transform is used to revert the data to real-space information (i.e., Fourier filtering). Alternatively, we can use another direct approach, real-space convolution, wherein the convolution kernel is small and comparable to the point spread function, which is equivalent to smoothing to reduce random noise in oversampled images. The Fourier transform of a smoothed image shows a decay in the contrast transfer at high frequencies.

In the actual data processing, *n*_*k*_ row vectors of $\:{\varvec{H}}^{\left(i\right)}$ are refolded as a set of maps $\:{{\varvec{h}}_{\varvec{k}}}^{\left(i\right)}$ (*k* = 0,1,…,*n*_*k*_−1). Then, we perform the constraint procedures as $\:{{\varvec{h}}_{\varvec{k}}}^{\left(i\right)}\leftarrow\:{{\varvec{h}}_{\varvec{k}}}^{\left(i\right)}\varvec{*}\varvec{g}$ for the set of maps, where $\:\varvec{g}$ is the assumed kernel, and $\:\varvec{*}$ represents convolution. The set of the convoluted maps is unfolded as row vectors of ***H***^(*i*)^, and we denote the whole procedure as $\:{\varvec{H}}^{\left(i\right)}\leftarrow\:{\left[{\varvec{H}}^{\left(i\right)}\right]}_{\text{H}}$. Figure 5 shows examples of the constraint on maps, and Fig. 5a shows a 3 × 3 convolution kernel based on a Gaussian distribution. The convolution kernel matches the expected spatial resolution, i.e., the steepness at the edges of the crystalline areas (see Fig. 10a). Figure 5b and c show example maps and their Fourier transforms before and after the constraint process, respectively. The convolution attenuated the high-frequency noise (outside the circle indicated by the dotted line in Fig. 5b). Although the 3 × 3 Gaussian distribution was used, the size and intensity profile of the kernel can be optimized for each experiment.

**Fig. 6:**
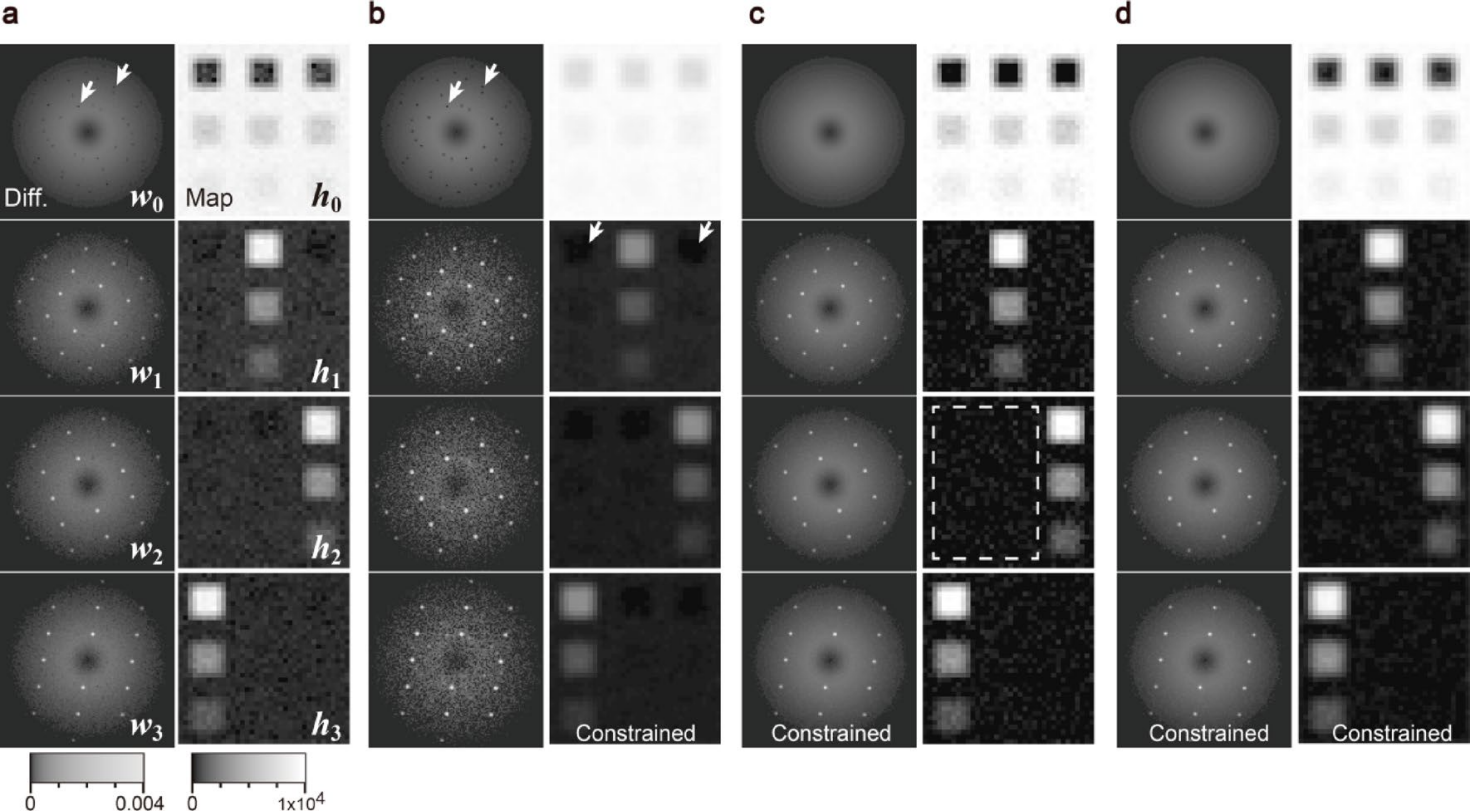
NMF results of simulated data under various constraint conditions. The correct number of components (*n*_*k*_ = 4) is assumed. **a** Primitive NMF (ALS). **b** Smoothing in maps. **c** Intensity continuity in diffractions. **d** NMF with constraints on both maps and diffractions. PCA results under the same condition are also given in the Supplementary Information (Fig. S3).

**Fig. 7:**
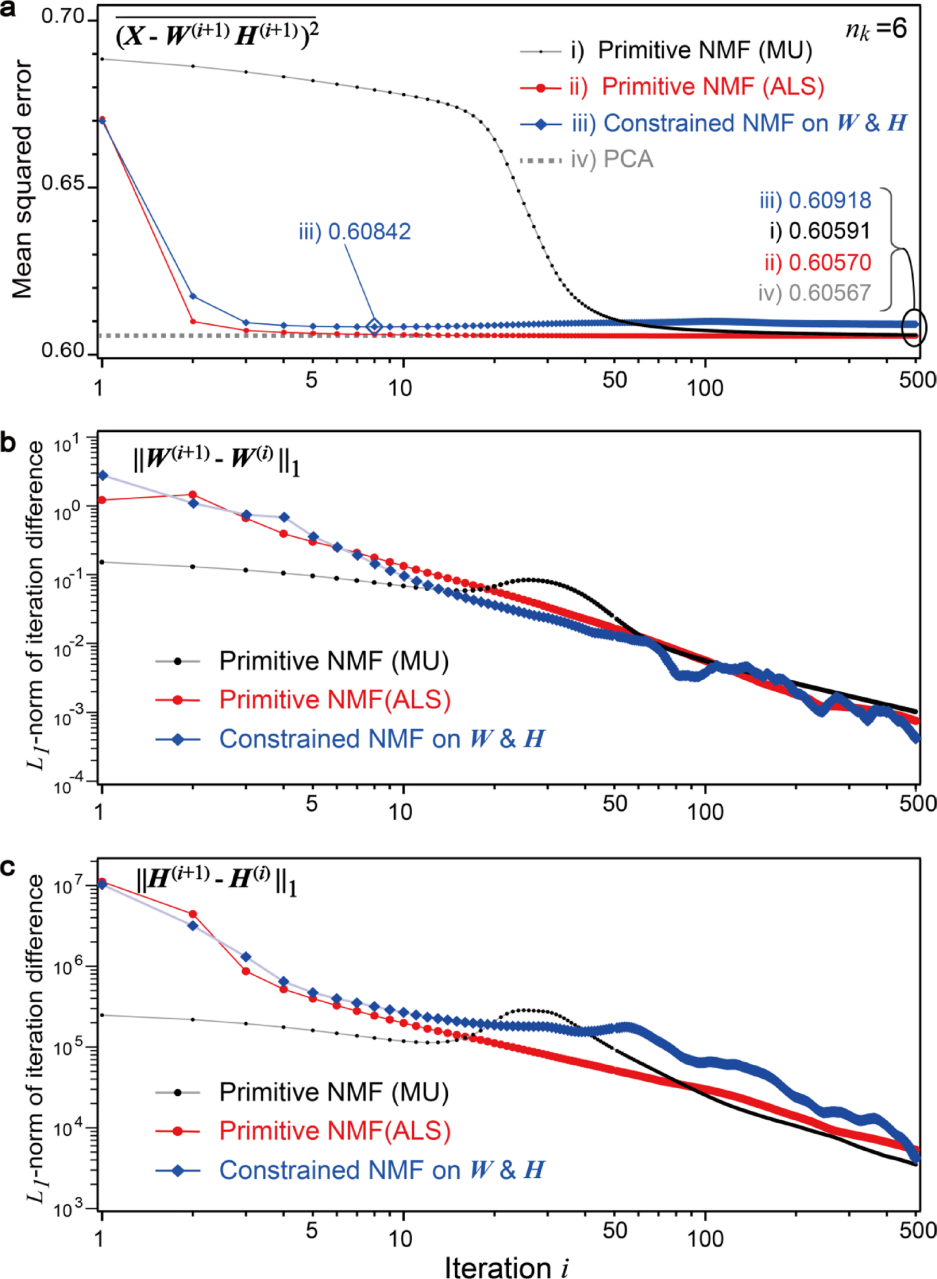
Convergence of various NMF algorithms as a function of iterations. **a** MSEs of a few NMF algorithms. The MSE of PCA is shown as a horizontal broken line. **b** and **c** *L*_*1*_-norms of the differences in the ***W***^**(*i*)**^ and ***H***^**(*i*)**^, respectively.

### NMF results of simulation data

We first demonstrate NMF on simulated 4D-STEM data, the structures of which are described in the Methods and Fig. 10. Figure 6 shows the NMF results under various constraint conditions: (a) primitive NMF (ALS), (b) smoothing in maps ($\:{[\:\cdot\:]}_{\text{H}}$), (c) intensity continuity in diffractions ($\:{[\:\cdot\:]}_{\text{W}}$), and (d) fully constrained for maps and diffractions (($\:{[\:\cdot\:]}_{\text{H}}$ and $\:{[\:\cdot\:]}_{\text{W}}$)). The correct number of components (*n*_*k*_ = 4) is assumed, and each result shows the minimum MSE from ten different random initializations. The primitive NMF (Fig. 6a) estimates one amorphous (*k* = 0) and three crystalline diffractions (*k* = 1, 2, 3), including the weak (1%) crystalline areas in the maps (see (iii), (vi), and (ix) in Fig. 10a). Many dark spot artifacts are observed in the amorphous diffraction pattern (arrows in ***w***_*0*_ in Fig. 6a). The positions of these dark spots correspond to those of the diffraction spots of the other components. The smoothing constraint on the maps (Fig. 6b) improves the signal-to-noise ratio in the maps, although artifacts still appear, such as the dark spots in the diffraction (***w***_***0***_) and the dark areas in the maps (***h***_***1***_,***h***_***2***_,***h***_***3***_), as indicated by the arrows. The continuity constraint on the diffractions (Fig. 6c) successfully eliminates these dark spots and areas; however, the noise in the maps is not negligible (see the rectangle in ***h***_2_ in Fig. 6c). Applying both constraints (Fig. 6d) can significantly reduce these artifacts.

**Fig. 8:**
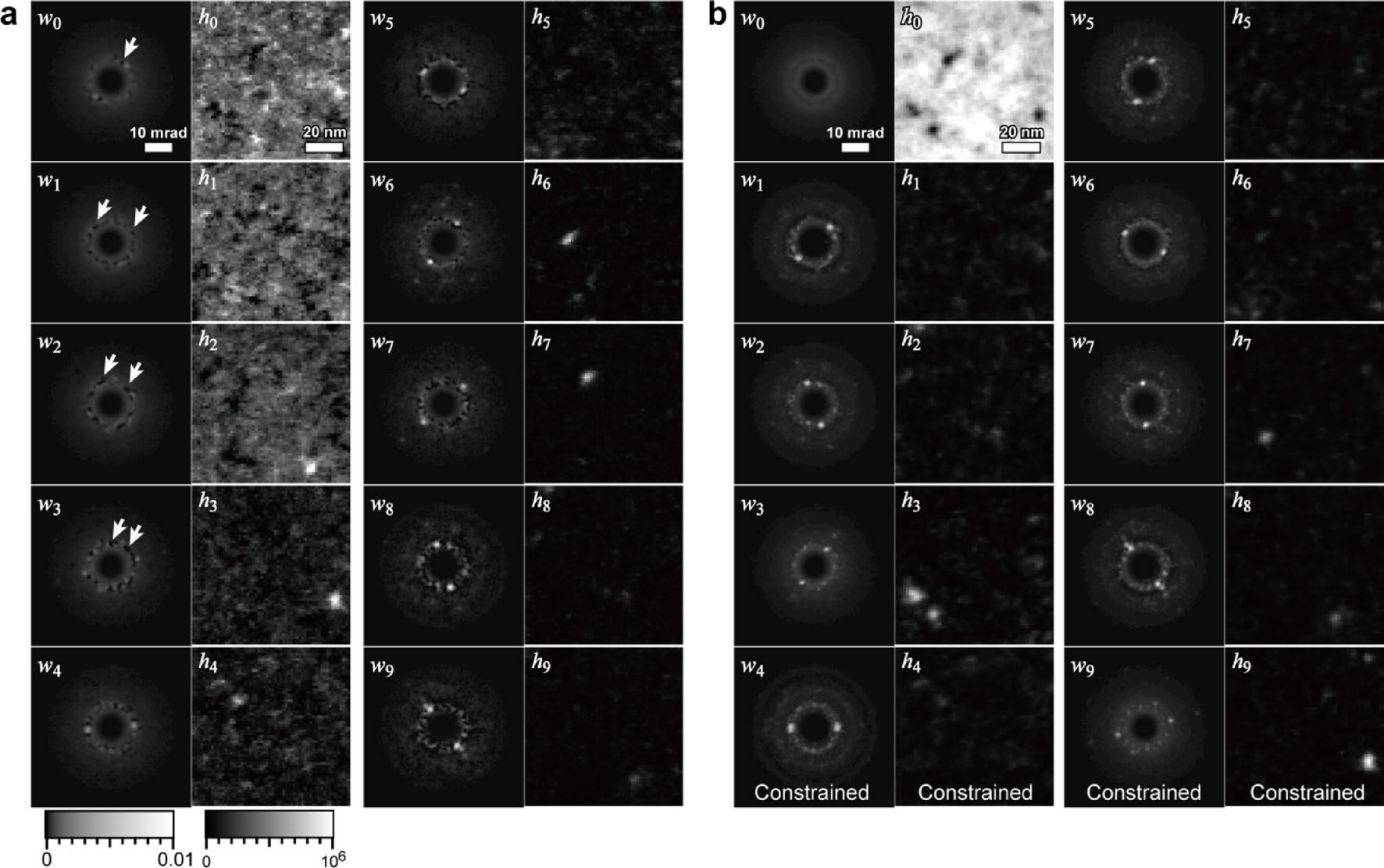
NMF results of 4D-STEM for ZrCuAl treated at high pressure and temperature. **a** Primitive NMF and **b** a fully constrained NMF. The number of components *n*_*k*_ is assumed to be 30, and the ten pairs of major components are shown. All 30 components are shown in **Fig. S5**. PCA results are also given in the Supplementary Information (Fig. S4).

The stopping criterion is important for general iterative algorithms. To confirm the convergence of the NMF iterations, we analyzed the MSEs and the differences in $\:{\varvec{W}}^{\left(i\right)}$ and $\:{\varvec{H}}^{\left(i\right)}$. Figure 7a shows the MSEs of the primitive NMF based on the MU and ALS algorithms and the constrained NMF as a function of iterations, assuming *n*_*k*_ = 6. In this case, the primitive NMF based on the ALS algorithms reduces the MSEs with fewer iterations than the MU algorithm. Even after 500 iterations, the MSE of the MU algorithm remains higher (0.60591) than that of the primitive ALS (0.60570). This faster convergence is a known advantage of ALS algorithms^28,30^, and we confirmed the convergence properties of our calculations with *n*_*k*_ = 4, 5, 6, 8, 10, and 12 (Fig. S2). The convergence of the NMF algorithms can be validated by comparing it to the MSE of the PCA, as indicated by the horizontal dashed line (0.60567). When domain-specific constraints are introduced, the converged MSE increases (0.60918 at *i* = 500). The MSE of the primitive NMF monotonically decreases through the iterations; however, that of the constrained NMF shows an initial minimum MSE within several iterations (0.60842 at *i* = 8) and then converges to a relatively high value (0.60918 at *i* = 500), as shown in Fig. 7a. Therefore, MSE is not always a suitable parameter for tracking convergence. We also calculated the *L*_*1*_-norms of the iteration differences $\:\left\| {\varvec{W}^{{(i + 1)}} - \varvec{W}^{{\left( i \right)}} } \right\|_{1}$ and $\:\left\| {\varvec{H}^{{(i + 1)}} - \varvec{H}^{{\left( i \right)}} } \right\|_{1}$, as shown in Fig. 7b and c, respectively. The *L*_*1*_-norms of the differences in all the NMF algorithms finally reach small values. In other words, all algorithms, including the constrained NMF, converge similarly to stationary points, as do the primitive MU and ALS algorithms. Thus, we can confirm their conversions by monitoring the differences, $\:\left\| {\varvec{W}^{{(i + 1)}} - \varvec{W}^{{\left( i \right)}} } \right\|_{1}$ and $\:\left\| {\varvec{H}^{{(i + 1)}} - \varvec{H}^{{\left( i \right)}} } \right\|_{1}$.

### NMF results and the hierarchical clustering of experimental data

Next, we apply the developed NMF technique to an actual material with an unknown number of components. The specimen was a ZrCuAl metallic glass annealed at 880 K under 5.5 GPa. The nanostructure of metallic glass has been analyzed using advanced electron microscopy techniques^35,36^. Because of the high-temperature, high-pressure treatment, nanometer-sized crystals precipitated in the amorphous matrix. The preliminary results have been reported elsewhere^16,37^. As shown in Fig. 8a and b, we performed the primitive and constrained NMFs (for both ***W*** and ***H***). The number of components *n*_*k*_ is assumed to be 30, and Fig. 8 shows ten pairs of diffractions and maps that indicate the high *L*_*2*_-norm of the maps. In other words, these components are dominant in the actual specimen. All 30 factorized diffractions and maps are provided in the Supplementary Information (Fig. S5).

**Fig. 9:**
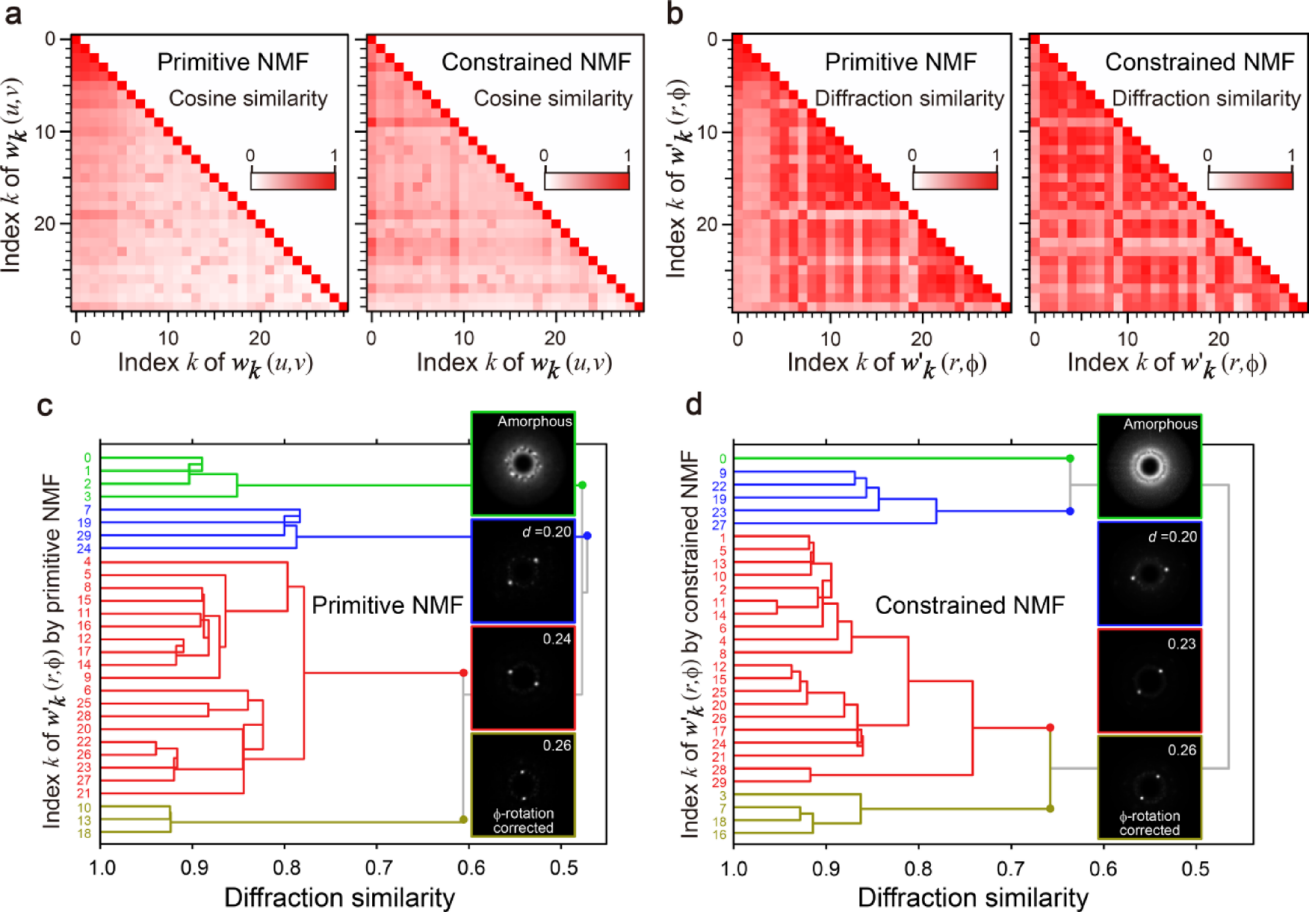
Cosine similarity, diffraction similarity, and hierarchical clustering of factorized diffractions. **a** Conventional cosine similarities of diffractions factorized by the primitive and constrained NMFs. **b** Diffraction similarities of the diffractions factorized by the primitive and constrained NMFs calculated using Eq. (10). **c** Dendrogram based on the diffraction similarity of the primitive NMF. Insets show integrated factorized diffractions after rotation correction. **d** Dendrogram of the constrained NMF.

In both the primitive and constrained NMF results (Fig. 8a and b), the lowest-index component ***w***_***0***_ does not show intense diffraction spots and corresponds to amorphous diffraction. In the case of primitive NMF, a few amorphous-like components appear (***w***_***0***_, ***w***_***1***_, ***w***_***2***_ and ***w***_***3***_ in Fig. 8a), but they suffer from dark spot artifacts, as indicated by the arrows. These diffractions are expected to be an identical crystallographic component, i.e., the amorphous matrix; however, because of the different dark spot artifacts, they are assigned as a few separate components. This is problematic because the multiple amorphous components of the artifact make it difficult to detect other crystalline components, even if we assume a large number of components *n*_*k*_. By contrast, the constrained NMF shows a single amorphous component, as shown in Fig. 8b, with no dark spots in the diffractions.

Both methods broadly identify similar crystalline precipitates. For example, the precipitates ***h***_**4**_ and ***h***_***9***_ of primitive NMF correspond to the precipitates ***h***_**4**_ and ***h***_**8**_ of constrained NMF, respectively. Note that the map ***h***_**4**_ of primitive NMF is noisier than ***h***_**4**_ of constrained NMF. This is consistent with the simulation results (Fig. 6), which demonstrate noise reduction by the constraint. The precipitate ***h***_***9***_ in constrained NMF resembles map ***h***_**3**_ of primitive NMF; however, ***h***_**3**_ of primitive NMF exhibits amorphous-like diffraction, which is discussed later (Fig. 9c). Consequently, this crystalline precipitate could be detected only by constrained NMF. These superior factorization properties of the constrained NMF can be quantitatively validated by similarity evaluation and hierarchical clustering, as described below.

**Fig. 10:**
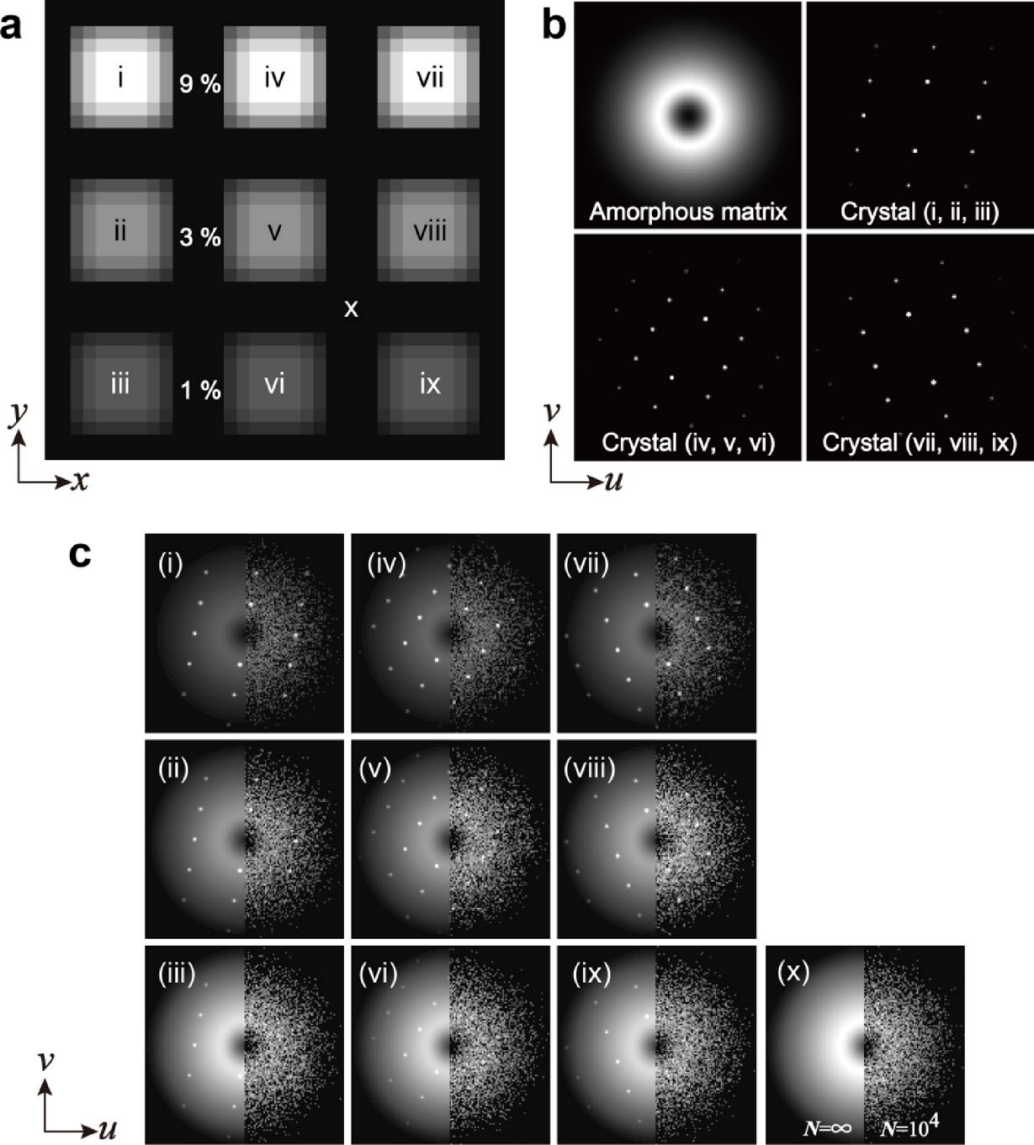
Simulated 4D-STEM data consisting of one amorphous and three crystalline components. **a** Real-space structure. **b** Four components of diffractions. **c** Diffraction examples from areas (i)–(x). 4D-STEM data with Poisson noise was used in this study.

To quantitatively compare the primitive and constrained NMFs, we evaluated the cosine similarities of each set of factorized diffractions. The cosine similarity, which is a standard measure in machine learning, is calculated using the following equation:

9 $$\:cosine\_similarity\:\left( {k_{1} ,k_{2} } \right) = \:\frac{{\left\langle {\varvec{W}\left( {:,k_{1} } \right),\varvec{W}(:,k_{2} )} \right\rangle }}{{\left\| {\varvec{W}(:,k_{1} )} \right\|_{F} \:\left\| {\varvec{W}(:,k_{2} )} \right\|_{F} }},$$

where $\:\varvec{W}(:,k)$ represents the *k*-th column vector of the matrix ***W***. Figure 9a shows the cosine similarities of the diffractions factorized by the primitive and constrained NMFs. The low-index diffractions (top-left corner) of the primitive NMF show high similarities, suggesting the same amorphous diffraction. However, the constrained NMF does not indicate other diffractions similar to the amorphous one. Thus, the appropriate constraints can eliminate the artifacts and multiple components resulting from artifacts, more clearly discriminating between the amorphous and crystalline areas.

The actual experimental data must include similar crystalline diffractions; however, the high-index crystalline diffractions show low values for the cosine similarity in both the primitive and constrained NMFs (Fig. 9a). This is because the similarity of rotated diffractions cannot be detected based on 1D measures, such as the cosine similarity or Euclidean distances, in standard machine learning techniques. By introducing 2D analytical techniques for electron microscopy, further information on the experimental data can be derived. Here, we define the diffraction similarity based on the cross-correlation of $\:r-\varphi\:$-transformed diffractions. In the case of cross-correlation, the similarity is given by the peak value, and the amount of pattern shift is measured by the peak position^38,39^. The diffraction similarity between two components *k*_*1*_ and *k*_*2*_ can be calculated using the following equation:

$$\:diffraction\_similarity\left({k}_{1},\:{k}_{2}\right)=\text{max}\left({{\varvec{w}}^{\varvec{{\prime}}}}_{k1}\left(r,\varphi\:\right)\star\:{{\varvec{w}}^{\varvec{{\prime}}}}_{k2}\left(r,\varphi\:\right)\right)\:$$

10 $$\:\:subject\:to\:\:r=0,$$

where $\:\star\:$ represents cross-correlation, and *r* = 0 is required to allow uniaxial shifts along the $\:\varphi\:$ axis of $\:{{\varvec{w}}^{\varvec{{\prime\:}}}}_{k}\left(r,\varphi\:\right)$, i.e., the $\:\varphi\:$-rotation of $\:{\varvec{w}}_{k}\left(u,v\right)$. The rotation angle can also be calculated using the uniaxial cross-correlation as follows:

$$\:diffraction\_rotation\left({k}_{1},\:{k}_{2}\right)=\underset{\varphi\:}{\text{arg\:max}}\left({{\varvec{w}}^{\varvec{{\prime}}}}_{k1}\left(r,\varphi\:\right)\star\:{{\varvec{w}}^{\varvec{{\prime}}}}_{k2}\left(r,\varphi\:\right)\right)\:\:$$

11 $$\:subject\:to\:\:r=0.$$

Using Eq. (10), we calculated the diffraction similarities of primitive and constrained NMF results (Fig. 9b). In contrast with the cosine similarity (Fig. 9a), the diffraction similarity can identify similar rotated diffractions among the high-index crystalline components. The $\:r-\varphi\:$ transformation and uniaxial cross-correlation are fundamental domain-specific knowledge in electron diffraction, and this combination is effective for elucidating the results of 4D-STEM.

Because the same diffractions, but rotated, are factorized as different components in all NMFs, clustering is required to categorize the factorized results based on diffraction physics^34^. The diffraction similarity mentioned above can be used as a substitute for conventional distances required in hierarchical clustering. Notably, standard techniques (e.g., *k*-means clustering) based on conventional distances are ineffective for experimental data consisting of randomly rotated patterns. Figure 9c and d show the dendrograms of the hierarchical clustering of the primitive and constrained NMF results, respectively. The diffraction similarity calculated by Eq. (11) was applied to compute the linkage matrices for SciPy^40^ using customized DigitalMicrograph scripts, and the dendrograms were plotted using NumPy^41^, SciPy, and Matplotlib^42^. During the hierarchical clustering, we also calculated averaged diffractions with $\:\varphi\:$-rotation corrections using Eq. (11). Four major clusters are found in both dendrograms, and each averaged diffraction is shown in the inset. The average crystalline diffractions in Fig. 9c and d show similar twin spots; however, their Bragg angles and corresponding distances *d* are different, resulting in the different clusters. The constrained NMF reveals only one amorphous component, whereas the primitive NMF derives four (*k* = 0, 1, 2, 3) because of the artifact (see the green lines in Fig. 9c and d). This clustering based on the diffraction similarity clarifies the difference between the primitive and constrained NMFs and is also an enhanced machine learning technique with domain-specific knowledge.

## Discussion

### Comparison with established software

In this study, the MU and ALS algorithms were implemented from scratch using custom DigitalMicrograph scripts. These custom scripts allowed us to optimize their functionality and monitor the convergence process. We also reproduced the standard NMF for 4D-STEM using scikit-learn, which is the most established package. The Python code for the scikit-learn NMF implementation on DigitalMicrograph is provided in Sect. 2 of the Supplementary Information (Listing S3), where several options, including regularization for both matrices ***W*** and ***H***, can be applied.

Regularization is a standard technique used in machine learning to avoid overfitting. We evaluated its effects on the simulated 4D-STEM data using scikit-learn, as shown in Fig. S1. We found that dark spot artifacts persisted even when regularization terms were applied. Although regularization is known to improve the generalization performance and mitigate the effect of noise, it does not eliminate the abovementioned artifacts. In terms of noise reduction, the smoothing constraint on the maps in this study is similar to a regularization term. However, our approach does not require hyperparameter optimization (e.g., the regularization amplitudes for ***W*** and ***H***), as the smoothing kernel is simply derived from electron microscopy knowledge.

### Versatility of the present constrained NMF

This constrained NMF is applicable to various analytical techniques, e.g., hyperspectral imaging for elemental mapping^43,44^. Spatial resolution, continuous intensity features, and nonnegativity are physically self-evident but have not been systematically exploited as constraints in factorizations. If the penalty terms in the cost function are differentiable (e.g., Tikhonov regularization), an exact update formula can be obtained, as in the MU algorithm. In various applications, actual domain-specific constraints are not always differentiable. However, such knowledge can be implemented using the present proposed scheme, which is based on the ALS algorithm.

The spatial resolution, i.e., the size and shape of the point spread function, depends on each experimental technique. For various analytical techniques, it is practical if a specific kernel function (e.g., a Gaussian, Lorentzian, or pseudo-Voigt function) can be applied without rebuilding the update equations for the NMF. In this study, the convolution kernel (see Fig. 5) is assumed based on the incident probe of STEM; however, it can also be optimized for the material properties. For example, if the distribution of the diffractions or spectra is expected to be spatially delocalized, we could set an extended convolution kernel for the maps according to the expected distribution. Additionally, the constrained NMF can be used for noise filtering, where the convolution kernel is intentionally made large.

In many machine learning techniques, hyperparameters must be optimized, and the present constrained NMF also requires some hyperparameters such as a convolution kernel and the number of components. From a practical point of view, it is convenient when less computation is required to optimize the hyperparameters themselves. The convolution kernel can be reasonably prepared based on domain-specific knowledge, i.e., expected spatial resolution and scanning step (see the Methods). The number of components *n*_*k*_ is a critical hyperparameter in all NMF algorithms. It is reported that the sufficient number of components *n*_*k*_ could be speculated by comparing the MSEs of PCA and NMF^12,13,16^. Even if the number of components assumed in NMF is set larger than the actual number, our procedure yields integrated components through hierarchical clustering. Therefore, this method can be considered robust with respect to hyperparameter optimization.

Although the proposed scheme is heuristic, it is a practical solver with superior versatility for various constraints. This scheme could provide a new solution for materials scientists who use off-the-shelf machine learning software without incorporating their domain-specific knowledge and have been troubled by artifacts.

## Methods

### Simulated 4D-STEM data

We prepared simulated 4D-STEM data consisting of one amorphous and three different crystalline diffractions, as shown in Fig. 10b (the number of components *n*_*k*_ is four) with dimensions (*n*_*x*_, *n*_*y*_, *n*_*u*_, *n*_*v*_) = (36, 36, 128, 128). The simulated data in real space (*x*,* y*) included nine crystalline areas (about 6 × 6 pixels each) in an amorphous matrix (Fig. 10a), where the interface between the amorphous matrix and crystalline areas was gradually changed. The crystallinity ratios of the nine areas varied between 9%, 3%, and 1%. Examples of diffraction at each single position are shown in Fig. 10c; the left halves represent ideal diffractions, and the right halves represent the simulated data with quantum noise, which was used in this study. The quantum noise was implemented based on the Poisson distribution of the number of electrons, *N* = 10^4^, in each diffraction. This condition is similar to that obtained with a probe current of 2 pA and an exposure time of 1 ms, which are practical experimental settings. As shown in Fig. 10c, diffraction spots were visible when the crystalline-to-amorphous ratio was 9% (i, iv, vii) and 3% (ii, v, viii); however, diffraction spots were difficult to recognize when the ratio was 1% (iii, vi, ix) because of severe quantum noise. This simulated 4D-STEM data was mathematically generated using DigitalMicrograph custom scripts based on the four normalized diffractions (Fig. 10b) and the distributions in real space (Fig. 10a). Poisson noise was randomly implemented on all diffractions.

### 4D-STEM experiments with a metallic glass specimen

We analyzed a metallic glass Zr_50_Cu_40_Al_10_ specimen subjected to a high-pressure (5.5 GPa) and high-temperature (880 K) treatment. The structural and mechanical properties of the specimens are detailed in our previous report^37^. A specimen for 4D-STEM was prepared by Ar ion milling (PIPS-II, Gatan) at 2 kV or less. We performed a 4D-STEM experiment using an electron microscope (Titan, Thermo Fisher Scientific) at an accelerating voltage of 300 kV (wavelength *λ* = 2.0 pm). The 4D-STEM data were obtained from an 87 × 87 nm^2^ area using a 1.5 nm scan step (58 × 58 pixels) and a diffraction of 128 × 128 pixels (i.e., $\:{I}_{4D}\in\:{\mathbb{R}}^{58\times\:58\times\:128\times\:128}$). We realized a small convergence semi-angle of 0.5 mrad using a small aperture diameter of 0.5 μm (i.e., high angular resolution), and we could clearly distinguish crystalline spots from the amorphous diffuse rings. The spatial resolution of the present 4D-STEM experiment depended on the diffraction limit, and the probe had a full width at half maximum of 2 nm. The scan step of 1.5 nm was a slight oversampling condition for the incident probe. Based on the estimated spatial resolution and the scan step in the experiment, we set the 3 × 3 Gaussian convolution kernel for smoothing in the constraint on map $\:{[\:\cdot\:]}_{\text{H}}$, which was similar for the simulated data (Fig. 5a). Diffractions were acquired with an exposure time of 10 ms using a charge-coupled device detector (US1000 series, Gatan), and their intensities were converted into the number of electrons.

The experimental data included subtle noise from the detection system and comparable quantum noise due to the limited number of electrons captured per pixel. Typically, hundreds of electrons are involved in each pixel, which can contain tens of a percent of quantum noise according to the Poisson distribution. Although no normalization or denoising was applied, minimal data preprocessing was performed prior to NMF. Each diffraction pattern was accompanied by an intense direct spot at the center, and this high-intensity area became dominant when calculating the MSE; however, the direct spot is insensitive to the crystal structure. We therefore used a mask to cover the intense direct spot to select the structure-sensitive area (see Fig. 2).

## Supplementary Information

Below is the link to the electronic supplementary material.

Supplementary Material 1

## Data Availability

The datasets generated during this study are available from the corresponding author upon reasonable request.
