# Supplementary material for: Nonnegative matrix factorization incorporating domain specific constraints for four dimensional scanning transmission electron microscopy
Source: Sci Rep. 2025 Nov 7;15:39143. doi: 10.1038/s41598-025-23541-7 (PMC12595046; doi:10.1038/s41598-025-23541-7)
Supplement: Supplementary file 1 — Supplementary Material 1 [file 41598_2025_23541_MOESM1_ESM.pdf]

Supplementary Information

Nonnegative matrix factorization incorporating domain specific constraints for four dimensional scanning transmission electron microscopy

Koji Kimoto*, Fumihiko Uesugi, Koji Harano, Jun Kikkawa, Ovidiu Cretu, Yuki Shibazaki, Motoki Shiga, and Atsushi Togo

*Corresponding author

Contents

S1. DigitalMicrograph scripts of two major algorithms for primitive nonnegative matrix factorization

S2. NMF for 4D-STEM using scikit-learn on DigitalMicrograph

S3. Detailed procedure for the constraint on diffraction

S4. Converged mean squared errors

S5. Principal component analysis (PCA) of simulated and experimental 4D-STEM data

S6. All NMF components (n_k = 30) of the experimental 4D-STEM data

S1. DigitalMicrograph scripts of two major algorithms for primitive nonnegative matrix factorization

Two major algorithms are used for primitive nonnegative matrix factorization (NMF): (a) multiplicative update (MU) and (b) alternating least squares (ALS). Here, we provide DigitalMicrograph (Gatan, Inc.) script codes for the two algorithms. The usage of the DigitalMicrograph scripting is given on the manufacturer's website (https://www.gatan.com/products/tem-analysis/gatan-microscopy-suite-software). In both codes, the square of the Frobenius norm of the difference is the cost function (objective function) $D = \frac{1}{2}\|\boldsymbol{X} - \boldsymbol{WH}\|_F{}^2$, where $\boldsymbol{X}$ is the experimental matrix, and $\boldsymbol{W}$ and $\boldsymbol{H}$ are lower-rank matrices than $\boldsymbol{X}$, as described in the main text. The matrices $\boldsymbol{W}$ and $\boldsymbol{H}$ represent the diffractions and maps, respectively, as shown in **Fig. 2** of the main text.

The basic equations and DigitalMicrograph script for the MU algorithm are as follows:

$$\boldsymbol{W} \leftarrow \boldsymbol{W} \circledast \boldsymbol{XH^T} \oslash \boldsymbol{WHH^T}\ , \quad \text{(S3)}$$

$$\boldsymbol{H} \leftarrow \boldsymbol{H} \circledast \boldsymbol{W^T X} \oslash \boldsymbol{W^T WH}\ , \quad \text{(S4)}$$

where $\circledast$ and $\oslash$ are elementwise multiplication and division, respectively.

Listing S1: DigitalMicrograph code snippet for NMF based on multiplicative update (MU) algorithm.

```
/* DigitalMicrograph script for multiplicative update (MU) NMF */
// Koji KIMOTO of NIMS
     H    = UniformRandom()
     W    = UniformRandom()
     HT   = MatrixTranspose(H)
     XHT  = MatrixMultiply(X, HT)
     WH   = MatrixMultiply(W, H)
     WHHT= MatrixMultiply(WH, HT)
     W    = W * XHT / WHHT            // Elementwise calculation
     WT   = MatrixTranspose(W)
     WTX  = MatrixMultiply(WT, X)
     WTW  = MatrixMultiply(WT, W)
     WTWH= MatrixMultiply(WTW, H)
     H    = H * WTX / WTWH            // Elementwise calculation
     MSE  = MeanSquare(X - MatrixMultiply(W,H))
```

The basic equations and a DigitalMicrograph script for the ALS algorithm are as follows:

$$\boldsymbol{W} \leftarrow [(\boldsymbol{XH^T})(\boldsymbol{HH^T})^{-1}]_+\ , \qquad \text{(S1)}$$

$$\boldsymbol{H} \leftarrow [(\boldsymbol{W^T W})^{-1}(\boldsymbol{W^T X})]_+\ , \qquad \text{(S2)}$$

where $[\,\cdot\,]_+$ represents the nonnegativity constraint.

Listing S2: DigitalMicrograph code snippet for NMF based on alternating least squares (ALS) algorithm.

```
/* DigitalMicrograph script for alternating least squares (ALS) NMF */
// UESUGI and KIMOTO of NIMS
     H    = UniformRandom()
     HT   = MatrixTranspose(H)
     HHT  = MatrixMultiply(H, HT)
     IHHT= MatrixInverse(HHT)
     XHT  = MatrixMultiply(X, HT)
     W    = MatrixMultiply(XHT, IHHT)
     W    = tert(W<0, 0, W)          // Nonnegativity constraint
     WT   = MatrixTranspose(W)
     WTW  = MatrixMultiply(WT, W)
     IWTW= MatrixInverse(WTW)
     WTX  = MatrixMultiply(WT, X)
     H    = MatrixMultiply(IWTW, WTX)
     H    = tert(H<0, 0, H)          // Nonnegativity constraint
     MSE  = MeanSquare(X - MatrixMultiply(W, H))
```

The computational loads of both codes are similar. Although the ALS algorithm involves the calculations of the inverse matrices, their computational cost is minimal because the matrices are small, $\boldsymbol{W}^{\boldsymbol{T}}\boldsymbol{W}$, $\boldsymbol{H}\boldsymbol{H}^{\boldsymbol{T}} \in \mathbb{R}_{+}^{n_k \times n_k}$.

S2. NMF for 4D-STEM using scikit-learn on DigitalMicrograph

Python libraries (e.g., SciPy, NumPy, and scikit-learn) can be used on DigitalMicrograph, and the 4D-STEM data in the DigitalMicrograph format (*.dm4) can be directly processed using these libraries. The following is the Python code for the NMF. Note the different order of the coordinates (*x, y, u, v*) of the 4D-STEM data.

Listing S3: Python script for NMF for DigitalMicrograph 4D-STEM data using scikit-learn.

```
# Scikit-learn NMF for 4D-STEM through Python on DigitalMicrograph
# Koji KIMOTO of NIMS
import DigitalMicrograph as DM
import numpy as np
import sys
import joblib
import scipy
import sklearn
# Get data array of frontmost image
SIdata = DM.GetFrontImage().GetNumArray()
nChannelsV = SIdata.shape[0]
nChannelsU = SIdata.shape[1]
nChannelsY = SIdata.shape[2]
nChannelsX = SIdata.shape[3]
nChannels  = nChannelsV * nChannelsU
nPoints    = nChannelsY * nChannelsX
# Unfolding
data = SIdata.reshape( nChannels, nPoints ).transpose()
# Parameters
from sklearn.decomposition import NMF
num_comp   = 4         # Assumed number of components
str_init   = 'random' # Initialization
str_solver = 'cd'      # Coordinate Descent(cd), Multiplicative Update
 (mu)
num_tol    = 1e-4      # Tolerance of stopping
num_maxi   = 3000      # Maximum iterations
alpha_W0   = 0.1       # Regularization for W
alpha_H0   = 0.1       # Regularization for H
print("\nNMF ")
print("Components\t Initialization\t Solver\t Tolerance\t
 MaxInteration\t Alpha_W\t Alpha_H")
print(num_comp, "\t\t", str_init,"\t\t", str_solver,"\t\t",
 num_tol,"\t\t", num_maxi,"\t\t", alpha_W0,"\t\t", alpha_H0)
# NMF
estimator = NMF(n_components=num_comp, init=str_init, solver=str_solver,
 tol=num_tol, max_iter=num_maxi, alpha_W=alpha_W0, alpha_H=alpha_H0)
# H refolding
arr_H = estimator.fit_transform(data)
asStackH = ( SIdata.shape[2] , SIdata.shape[3], num_comp )
resultStackH = DM.CreateImage(arr_H.reshape(asStackH ).transpose(2,0,1).
 copy())
resultStackH.SetName("Map")
resultStackH.ShowImage()
# W refolding
arr_W = estimator.components_
asStackW = ( num_comp, nChannelsU, nChannelsV  )
resultStackW = DM.CreateImage( arr_W.reshape( asStackW ).copy() )
resultStackW.SetName("Diff.")
```

```
resultStackW.ShowImage()
# Mean squared error (MSE)
HW  = np.matmul(arr_H,arr_W)
XHW = data - HW
XHW2 = XHW**2
mse = XHW2.sum()
mse = mse/(nChannels * nPoints)
print("Mean squared error: ", mse)
del resultStackH
del resultStackW
```

Figures S1a and S1b show the NMF results using this Python script, in which the parameters for NMF are equal, except for the regularizations for $\boldsymbol{W}$ and $\boldsymbol{H}$. Dark spot artifacts (white arrows) are observed in the diffractions, even in the case of regularized NMF.

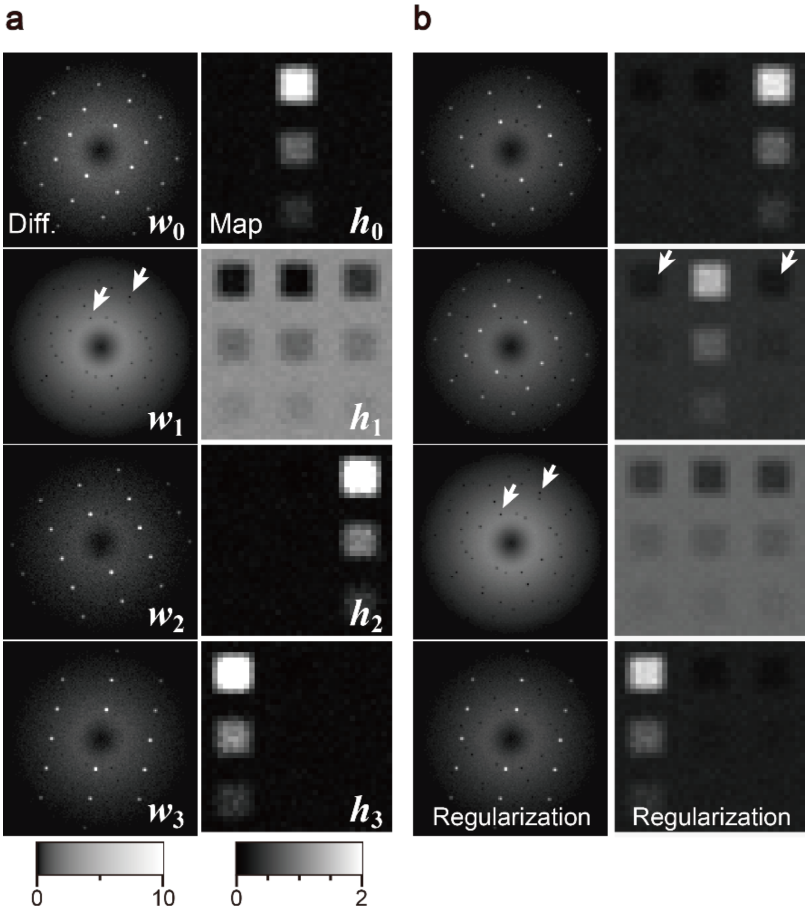

Fig. S1: NMF results of the simulated 4D-STEM data analysed using scikit-learn library. Parameters for NMF are given in the abovementioned Python code. **a** NMF without regularization. **b** NMF with regularizations (0.1 for both ***W*** and ***H***). White arrows indicate the artifacts.

S3. Detailed procedure for the constraint on diffraction

The constraint on diffraction is outlined in the main text and illustrated in Fig. 4. Here, we present the DigitalMicrograph script used to remove the downward-convex peak in diffractions. The following script can be used without additional code. DigitalMicrograph's intrinsic variables (`icol, irow, and iradius`) and functions (`warp(), tert()`) allow us to perform the desired image processing with minimal descriptions.

Listing S4: DigitalMicrograph script for constraint on diffraction, $[\cdot]_W$.

```
/* DigitalMicrograph script for downward-convex peak correction */
// Koji KIMOTO of NIMS
image ColumnOutlierEraser(image imgIn)
{
number thresholdfactor = 2  // threshold = mean *2
image imgOut=imageclone(imgIn)
number sizeX, sizeY
GetSize(imgIn, sizeX, sizeY)
imgOut := imageclone(imgIn)
image columnI
    for(number i=0; i<sizeX ;i++)
    {
    columnI = imgIn[0,i,sizeY,i+1]
    number mean = mean(columnI)
    columnI = tert(columnI<(mean*thresholdfactor),columnI,mean)
    imgOut[0,i,sizeY,i+1] = columnI[0,0,sizeY,1]
    }
return imgOut
}

image RotationalAvergeWOO(image img)
{
number samplingfactor = 8 // Rotation sampling factor
number sizeX,sizeY
GetSize(img, sizeX, sizeY)
number size = min(sizex, sizeY)
number sampling = (size/2)*samplingfactor
image  imgRPhi := CreateFloatImage("r-phi transformed", size/2, sampling)
number k = 2*pi()/sampling
imgRPhi = warp(img,icol*sin(irow*k)+sizeX/2,icol*cos(irow*k)+sizeY/2)
imgRPhi = ColumnOutlierEraser(imgRPhi)
image  img1DProj := CreateFloatImage("1D projection", size/2, 1 )
img1DProj[icol,0] += imgRPhi
img1DProj /= sampling
image imgOut = imageclone(img)
imgOut = warp( img1DProj, iradius, 0 )
return imgOut
}

// Main
image img:=GetFrontImage()
image imgRAWOO  = imageclone(img)
SetName(imgRAWOO, "Rotational average without outliers")
imgRAWOO = RotationalAvergeWOO(img)
imgRAWOO /= sum(imgRAWOO)
ShowImage(imgRAWOO)
image imgDarkPeak = imageclone(img)
```

```
setname(imgDarkPeak, "Downward-convex peaks")
imgDarkPeak = - img + imgRAWOO
imgDarkPeak = tert(imgDarkPeak>0, imgDarkPeak, 0)
ShowImage(imgDarkPeak)
image imgNDCP = imageclone(img)
SetName(imgNDCP, "No downward-convex peak")
imgNDCP = img + imgDarkPeak
ShowImage(imgNDCP)
```

S4. Converged mean squared errors

The mean squared errors (MSEs) decrease with the increasing number of elements. Below, we have plotted the MSEs of the principal component analysis (PCA) and primitive nonnegative matrix factorization (NMF) based on the alternating least squares (ALS) and multiplicative update (MU) algorithms as functions of the number of assumed components (Fig. S2). The simulated 4D-STEM data were used. These calculations used a maximum of 500 iterations. The MSEs of ALS were always closer to that of PCA than they were to the MSEs of MU.

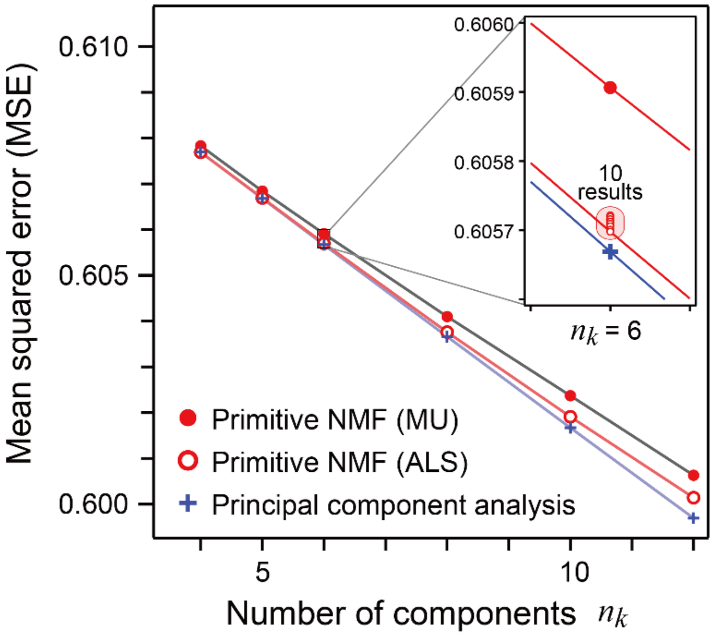

Fig. S2: MSEs of primitive NMF algorithms and principal component analysis as a function of assumed number of components n_k. All the iterations for the primitive NMFs are 500. MU and ALS algorithms were used in primitive NMF.

S5. Principal component analysis (PCA) of simulated and experimental 4D-STEM data

Figure S3a shows the first four components of the simulated data processed using principal component analysis (PCA). Because the maps and diffractions contain negative areas and peaks, they cannot be interpreted. **Figure S3b** shows the Varimax rotation of the PCA results. Although the negative peaks in the diffractions have disappeared, negative values remain in the map, and downward-convex peaks appear in the diffractions (see the arrows in **Fig. S3b**).

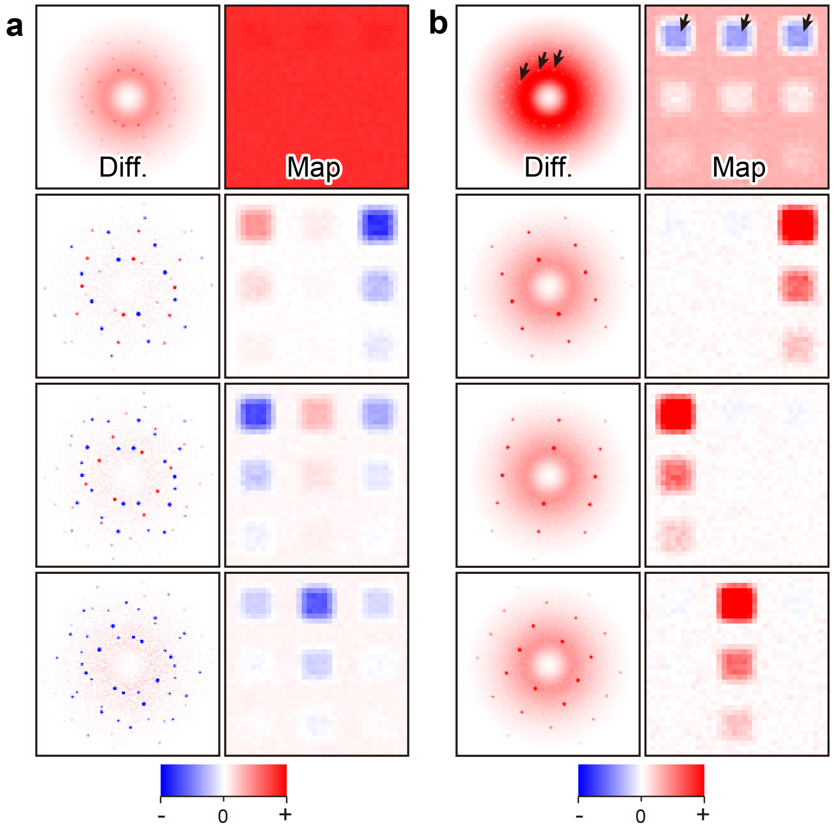

Fig. S3: PCA of simulated 4D-STEM data. a Four principal components of standard PCA. **b** Varimax-rotated results.

The results of the PCA of the experimental data for metallic glass are shown in **Fig. S4**. In the scree plot (**Fig. S4a**), no so-called elbow appears, making it difficult to identify the number of elements. The electron diffractions and maps extracted as principal components are shown in **Fig. S4b**. The components cannot be interpreted because of the negative values.

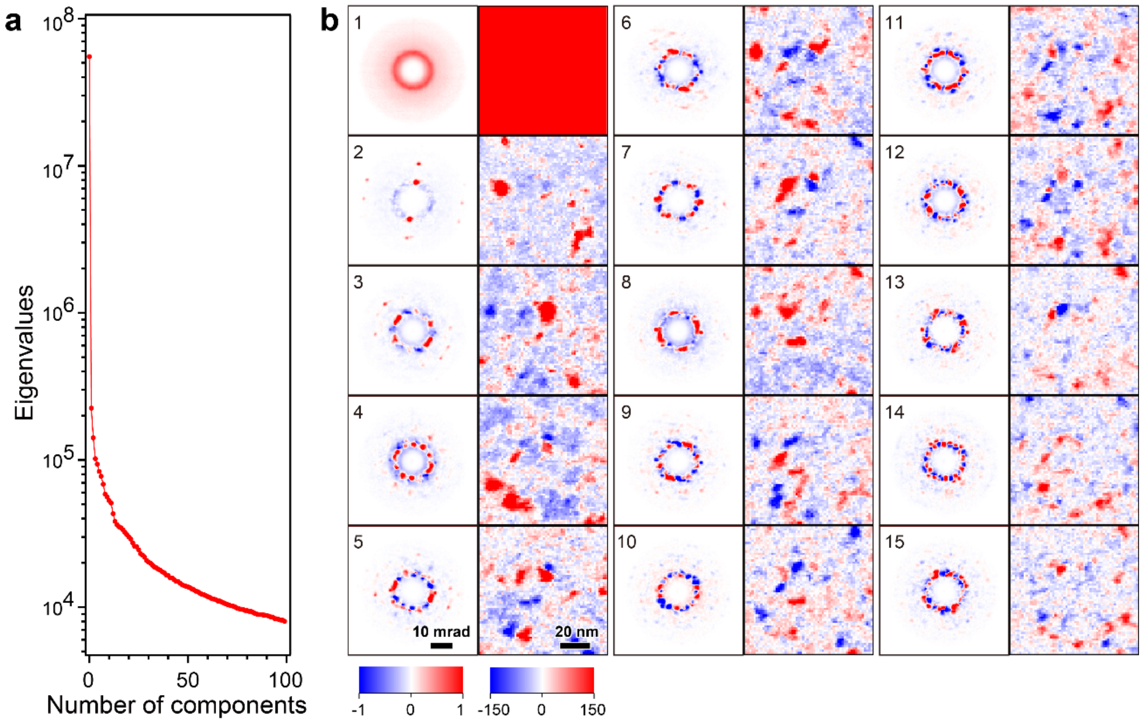

Fig. S4: PCA of experimental 4D-STEM data of high-pressure-, high-temperature-treated ZrCuAl. a Scree plot. **b** Diffractions and maps of the first fifteen components.

S6. All NMF components (n_k = 30) of the experimental 4D-STEM data

In the main text and **Fig. 8**, we show some of the NMF results for the experimental 4D-STEM data. **Figures S5a** and **S5b** show all the factorized results. The number of components n_k was assumed to be 30.

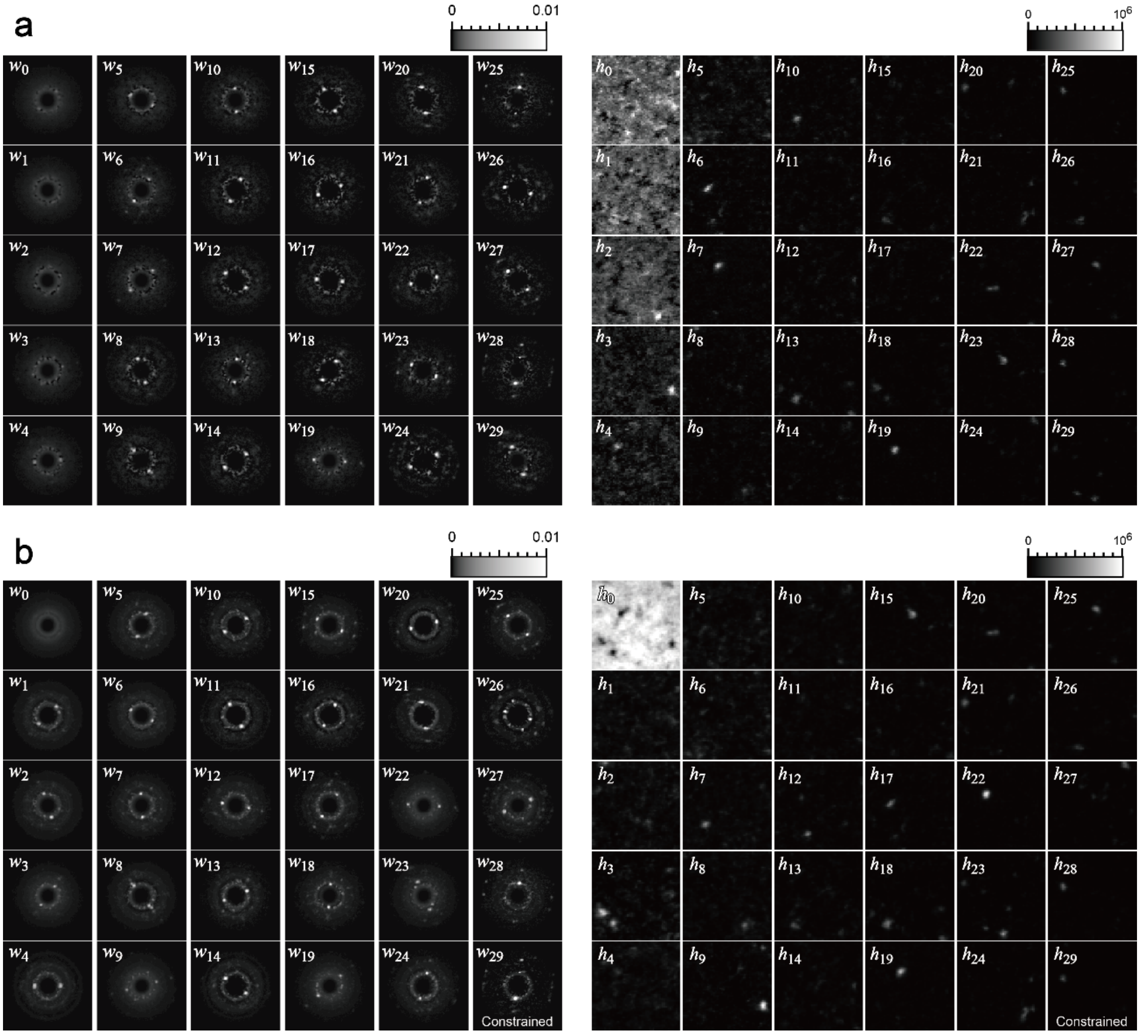

Fig. S5: NMF of experimental data on ZrCuAl from 4D-STEM. a and **b** All diffractions and maps obtained using **a** the primitive NMF and **b** the fully constrained NMF.
